# Temperature-induced changes of HtrA2(Omi) protease activity and structure

**DOI:** 10.1007/s12192-012-0355-1

**Published:** 2012-08-01

**Authors:** Dorota Zurawa-Janicka, Miroslaw Jarzab, Agnieszka Polit, Joanna Skorko-Glonek, Adam Lesner, Agata Gitlin, Artur Gieldon, Jerzy Ciarkowski, Przemyslaw Glaza, Agnieszka Lubomska, Barbara Lipinska

**Affiliations:** 1 Present Address: Department of Biochemistry, University of Gdansk, Kladki 24, 80-952 Gdansk, Poland; 2 Present Address: Department of Biochemistry, Biophysics and Biotechnology, Jagiellonian University, Gronostajowa 7, 30-387 Krakow, Poland; 3 Present Address: Faculty of Chemistry, University of Gdańsk, Sobieskiego 18/19, 80-952 Gdansk, Poland

**Keywords:** HtrA2 human protease, Temperature dependence of HtrA2 activity, HtrA2 structural changes, Fluorescence spectroscopy, Acrylamide quenching, Trp mutants

## Abstract

HtrA2(Omi), belonging to the high-temperature requirement A (HtrA) family of stress proteins, is involved in the maintenance of mitochondrial homeostasis and in the stimulation of apoptosis, as well as in cancer and neurodegenerative disorders. The protein comprises a serine protease domain and a postsynaptic density of 95 kDa, disk large, and zonula occludens 1 (PDZ) regulatory domain and functions both as a protease and a chaperone. Based on the crystal structure of the HtrA2 inactive trimer, it has been proposed that PDZ domains restrict substrate access to the protease domain and that during protease activation there is a significant conformational change at the PDZ–protease interface, which removes the inhibitory effect of PDZ from the active site. The crystal structure of the HtrA2 active form is not available yet. HtrA2 activity markedly increases with temperature. To understand the molecular basis of this increase in activity, we monitored the temperature-induced structural changes using a set of single-Trp HtrA2 mutants with Trps located at the PDZ–protease interface. The accessibility of each Trp to aqueous medium was assessed by fluorescence quenching, and these results, in combination with mean fluorescence lifetimes and wavelength emission maxima, indicate that upon an increase in temperature the HtrA2 structure relaxes, the PDZ–protease interface becomes more exposed to the solvent, and significant conformational changes involving both domains occur at and above 30 °C. This conclusion correlates well with temperature-dependent changes of HtrA2 proteolytic activity and the effect of amino acid substitutions (V226K and R432L) located at the domain interface, on HtrA2 activity. Our results experimentally support the model of HtrA2 activation and provide an insight into the mechanism of temperature-induced changes in HtrA2 structure.

**Electronic supplementary material:**

The online version of this article (doi:10.1007/s12192-012-0355-1) contains supplementary material, which is available to authorized users.

## Introduction

It is of biological importance that the hydrolytic enzymes catalyzing protein degradation should exhibit their activity at the proper cellular or extracellular location and under specific physiological conditions only. Usually these enzymes remain in inactive forms until their function is required. This rule applies to the high-temperature requirement A (HtrA) family of proteins whose members are very well conserved in evolution. Generally, they have proteolytic and chaperone activity and recognize unfolded proteins with exposed hydrophobic stretches. They play very important roles in the cells, and their functions are usually implicated in the protection of organisms from the effects of stressful conditions (for example heat or oxidative shock), which may cause aberrations in protein structure. To date, four human members of this family, HtrA1–HtrA4, have been identified, and it has been shown that they participate in protein quality control, regulation of cell proliferation, cell migration and fate (recently reviewed by Clausen et al. 2011; Singh et al. 2011).

The HtrA2(Omi) protease in physiological conditions serves as a protein quality control factor in mitochondria. Loss of HtrA2 causes an accumulation of unfolded proteins in mitochondria, dysfunction of the mitochondrial respiration, and generation of reactive oxygen species and contributes to cell death (Krick et al. 2008; Moisoi et al. 2009). In stressful conditions, HtrA2 may switch from a protector into a proapoptotic factor. Following the apoptotic stimuli, HtrA2 is released from the mitochondrium to the cytosol where it interacts with and degrades the inhibition of apoptosis proteins (IAPs). The neutralization of IAPs contributes to the induction of apoptosis. HtrA2 can also trigger cell death in a caspase-independent manner (reviewed by Vande Walle et al. 2008; Bhuiyan and Fukunaga 2009; Żurawa-Janicka et al. 2010). Regulation of cell death is linked with cancer development—indeed, there are many indications of HtrA2 involvement in oncogenesis (reviewed by Żurawa-Janicka et al. 2010; Hartkamp et al. 2010). Lately, dysfunctional HtrA2 has been implicated in the pathogenesis of several neurodegenerative disorders. In Parkinson’s disease, HtrA2 plays an important role in mitochondrial quality control (reviewed by Bhuiyan and Fukunaga 2009; Dagda and Chu 2009; de Castro et al. 2011), and it may contribute to Alzheimer’s disease through its interactions with presenilin (Gupta et al. 2004; Behbahani et al. 2010), amyloid precursor protein (Park et al. 2006), or amyloid-β peptide (Park et al. 2004; Kooistra et al. 2009).

All these facts lead to the assumption that HtrA2 could be a novel target in cancer therapy (reviewed by Chien et al. 2009; Żurawa-Janicka et al. 2010) or in the therapy of neurodegenerative disorders (reviewed by Bhuiyan and Fukunaga 2009). However, before using HtrA2 as a therapeutic target, its molecular mechanism of activation should be fully understood.

A considerable amount of research has been directed toward understanding the mechanism of action of HtrA proteases. The HtrA family of serine proteases can be distinguished from other serine proteases by sequence homology and also by the presence of at least one C-terminal PDZ domain, where PDZ stands for postsynaptic density of 95 kDa, disk large, and zonula occludens 1 domain. The N-terminal domain of HtrAs is variable, and it may contain signal and regulatory sequences. The protease domain is of the chymotrypsin type and is composed of two six-stranded β-barrels; the active site consisting of the amino acid triad His–Asp–Ser is located at the interface of the two perpendicularly arranged β-barrels. Apart from the β-strands, the domain contains several loops which are named, according to the chymotrypsin nomenclature, LA, L1, L2, L3, and LD and which are important for proteolytic activity and its regulation (reviewed by Clausen et al. 2011). The PDZ domains recognize and bind specific hydrophobic sequences in the C-termini of substrates or regulatory peptides and thus participate in the regulation of HtrA catalytic activity. They may also participate in the assembly of the oligomeric structures of HtrA proteins. The described HtrA monomers form higher-order oligomeric structures sharing a common basic building block. The common structural unit is a funnel/pyramid-shaped trimer consisting of protease domains, which form the central core and outward-protruding PDZ domains. In some HtrAs, e.g., in the *Escherichia coli* HtrA(DegP), this trimeric unit may further oligomerize. At low temperatures or in the absence of substrate, these proteins adopt inactive conformations which, according to the crystal structures, are characterized by the improper organization of active site residues and/or a restricted access to the catalytic triad. Hence, these proteins must become activated to perform their functions (reviewed by Krojer et al. 2008, 2010; Clausen et al. 2011; Singh et al. 2011).

To date, crystal structures of the active and inactive conformations were obtained for the *E. coli* HtrA(DegP) (Krojer et al. 2002, 2008; Kim et al. 2011), *E. coli* DegS (Wilken et al. 2004; Zeth 2004; Sohn and Sauer 2009), and human HtrA1 (Truebestein et al. 2011; Eigenbrot et al. 2012), which at the amino acid level bear 53, 51, and 74 % similarity to HtrA2, respectively (taking into consideration the protease and PDZ domains). HtrA(DegP) has two PDZ domains, while DegS and HtrA1 have single PDZ modules. In the resting (inactive) state, HtrA(DegP) exists as a hexamer formed by a staggered association of two trimeric rings. The proteolytic sites are hidden in a central cavity. The top and the bottom of the cavity are constructed by the six protease domains. The side walls are generated by the mobile PDZ domains, PDZ1 and PDZ2. In the hexamer, the active centers of HtrA(DegP) are in a catalytically incompetent state. The LA loops of one trimeric ring protrude into the active sites of the opposite ring, and there they interact with the loops L1 and L2, blocking access to the catalytic sites; furthermore, the active site does not have a proper conformation (Krojer et al. 2002). To gain activity, the molecule must undergo significant conformational changes, in particular disruption of the loop trio L1–L2–LA. Transition to a proteolytically active state is believed to occur in two major ways: (1) temperature-induced activation and (2) allosteric activation. Temperature has been shown to induce conformational changes within the HtrA(DegP) molecule. In particular, changes within the regulatory loops L1, L2, and LA have been demonstrated. A temperature shift induces exposition of the loops to solvent: the LA loop reacts first, followed by the remaining loops (Sobiecka-Szkatula et al. 2009). Allosteric activation is caused by a peptide long enough to bind to the active center and to the PDZ1 domain simultaneously (Kim et al. 2011). According to the current model (Clausen et al. 2011), binding of an appropriate peptide to the PDZ1 domain triggers the conformational changes of the whole HtrA(DegP) oligomer. First, peptide-induced remodeling of the binding cleft in PDZ1 affects the structure of a linker segment between the PDZ1 and PDZ2 domains, leading to dissociation of hexameric molecules into trimers. The trimers subsequently assemble into 12- or 24-mers. The reorganization of oligomers enables the PDZ1 domain to interact with the sensory loop L3 that in turn transmits the signal to the activation domain: the loops LD, L1, and L2. Additionally, the LA loop no longer interacts with the loops L1 and L2. The final effect is the formation of a proper active site and its accessibility. The *E. coli* DegS differs from HtrA(DegP), since it has only one PDZ domain and is a trimer in both the resting and active states. The PDZ domains of the oligomer function as inhibitory modules and form numerous contacts with the proteolytic domains. These interactions stabilize the inactive conformation where the active site triad and the oxyanion hole do not have the proper geometry for the catalysis (Wilken et al. 2004; Zeth 2004). DegS behaves as a classical allosteric enzyme and exists in tense (inactive) and relaxed (active) conformations which are in dynamic equilibrium (Sohn and Sauer 2009). A regulatory peptide acts as an allosteric activator. Its binding to the PDZ domain triggers a series of conformational changes at the interface of the PDZ and proteolytic domain and leads to the formation of a proper active center (Sohn et al. 2007, 2009, 2010; Sohn and Sauer 2009). HtrA1 forms a trimer resembling a flat disk. In the inactive state, the active-site triad and the oxyanion hole do not have a proper conformation, while the ligand-bound enzyme has a functional catalytic triad, oxyanion hole, and S1 specificity pocket. In this case, the PDZ domain is dispensable for activation since deletion of the PDZ domain does not affect the in vitro enzymatic activity of HtrA1 (Truebestein et al. 2011). Eigenbrot et al. (2012) showed that unliganded HtrA1 exists in two states, the active and inactive. Their results suggest a two-state equilibrium and a “conformational selection” model, in which substrate binds to the active conformer. Additionally, the crystal structures of the active and inactive forms of the proteolytic domain of *Thermotoga maritima* (HtrA_TM_) have been solved. In the resting state, the active site is covered by an α-helical lid formed by an LA loop. When temperature rises, the lid is lifted out and exposes the active site to the solvent, making it accessible to substrate. In this protein, the role of the PDZ domain is not known, since only the protease domain has been investigated (Kim et al. 2003, 2008). In summary, the activation scenarios among the HtrA proteins differ on many points.

HtrA2 is a trimeric protein, with a single PDZ domain per monomer. Solution of the HtrA2 crystal structure showed that it forms a pyramid-shaped homotrimer mediated by serine protease domains. The catalytic triad is formed by His198, Asp228, and Ser306 and is in a proper conformation for catalysis. The PDZ domain is linked to the protease domain via a flexible linker sequence and has a peptide-binding groove formed by the β14 and α7 structures; the groove contains the peptide-recognition motif, YIGV (at the positions 361–364). The peptide-binding pocket of the PDZ domain is buried in the interface between the PDZ and the protease domains, and access to the active site of serine protease is restricted by the PDZ domain. The PDZ domain packs against the protease domain through van der Waals contacts, and the hydrophobic residues on strands β11 and β12 of the protease domain interact with the hydrophobic residues from strand β14 and helix α5 of the PDZ domain (Li et al. 2002; Zhang et al. 2007). Based on this structure, which represents the resting form of HtrA2, Li et al. (2002) developed a model of HtrA2 activation. It suggests that the binding of a peptide to a hydrophobic groove of the PDZ domain leads to a significant conformational change at the PDZ–protease interface. This conformational change removes the inhibitory effect of PDZ from the active site, thus enhancing its activity. This model is supported by the fact that the PDZ-deleted HtrA2 variant is more active than the full-length protein (Li et al. 2002). It has been shown that peptides binding to the PDZ domain cause an increase in HtrA2 activity. Martins et al. (2003) selected a series of peptides binding to the isolated PDZ domain and found that the peptide biotin-GQYYFV-COOH (termed PDZ-Opt), which binds efficiently to PDZ, was able to stimulate HtrA2 activity measured with synthetic substrate peptide. Gupta et al. (2004) demonstrated that a peptide corresponding to the cytoplasmic C-terminal tail of presenilin 1 increased the proteolytic activity of HtrA2 toward the IAPs and β-casein. On the other hand, HtrA2 is highly dependent on temperature. Previous studies showed that it was up-regulated in mammalian cells in response to heat-shock-induced stress (Gray et al. 2000). Martins et al. (2003) observed that preincubation of HtrA2 at 42 °C resulted in increased proteolytic activity toward substrate peptide. Zhang and Chang (2004) found that HtrA2 activity toward casein increased rapidly with temperature; no changes in oligomerization occurred up to 55 °C, but significant change at the tertiary structure level was observed, using the near-UV circular dichroism (CD). As the heat-shock-treated HtrA2 could not be further stimulated by activating peptide and HtrA2 lacking PDZ could not be stimulated by heat shock, it has been postulated that both treatments might have a similar effect on the structure of HtrA2, resulting in displacement of the PDZ domain from the active site (Martins et al. 2003). The crystal structure of the active HtrA2 conformation is not yet known. Activation of HtrA2 resembles that of DegS due to the participation of PDZ domains in this process. However, while the PDZ domain stabilizes the inactive form in DegS, in HtrA2 it blocks the access of the substrate to the active center. Furthermore, there are no data indicating that DegS is activated by temperature. Additionally, in the resting state of protease, the HtrA2 active site is in a proper conformation, while in DegS it needs remodeling.

The aim of this work was to examine the putative conformational changes of HtrA2 expected to take place during the HtrA2 activation, in particular in the area of the interface between the protease and PDZ domains. To probe the events that occurred when the temperature was increased, we prepared single-tryptophan-containing mutants of HtrA to take advantage of the selective spectroscopic properties of Trp. By the aid of fluorescence spectroscopy, it was possible to detect significant changes in the protease and PDZ domains. The observed changes correlated well with the temperature-dependent pattern of stimulation of the HtrA proteolytic activity and indicated that at increased temperatures, the HtrA2 structure relaxes and the interface between the domains becomes more exposed to the solvent. The relaxation was further supported by demonstrating, using the dynamic light scattering (DLS) technique, that the HtrA2 particle size significantly increased with a rise in temperature. Furthermore, the point mutations V226K and R432L affected HtrA2 proteolytic activity in a manner consistent with the hypothesis that interaction of the PDZ domain with the protease domain changes upon activation.

## Materials and methods

### Materials

Fluorescent substrate Ala(Mca)IRRVSYSF-ANB-NH_2_ (where Mca is 7-methoxycoumarin-4-acetic acid and ANB-NH_2_ is amide of amino benzoic acid) whose sequence was based on substrate described by Martins et al. (2003) and the activating peptide: Acetyl-GQYYFV-COOH (Martins et al. 2003) was synthesized on solid support using Fmoc chemistry applying the procedure described earlier (Wysocka et al. 2010). The final product was removed from the support with simultaneous deprotection of the side chain groups. Finally, the crude product was analyzed on a reverse phase system using a ProSTAR HPLC s (Varian) and subjected to matrix-assisted laser desorption/ionization time-of-flight mass spectrometry to confirm its identity. Restriction enzymes and T4 ligase were purchased from Fermentas (Vilnius, Lithuania); primers used in site-directed mutagenesis were purchased from Proligo (Boulder, CO, USA). Fmoc-β-(7-methoxy-coumarin-4-yl)-Ala-OH was obtained from Bachem AG (Switzerland). Other chemicals were purchased from Sigma or Fluka (Poznan, Poland) and were of the highest quality and purity.

### Strains and plasmids

*E. coli* strain *E. coli* BL21(DE3) F^−^ *ompT hsdS*_*B*_(*r*_*B*_^−^ *m*_*B*_^−^) *gal dcm* was used for overproduction of HtrA2 proteins. The pJN1 plasmid (Żurawa-Janicka et al. 2008) was used as a source of *HtrA2* cDNA for cloning. The pDZ10 plasmid, carrying the wild-type mature *HtrA2* gene, and the pDZ5 plasmid, carrying *HtrA2* S306A (Ser306 → Ala substitution) were obtained in this work as described below. The remaining plasmids are pDZ5 or pDZ10 derivatives carrying variants of *HtrA2* gene: pDZ10 V226K (*HtrA2* V226K), pDZ5 V226K (*HtrA2* V226K S306A), pDZ5V226W(*HtrA2* V226W S306A), pDZ10 V226W (*HtrA2* V226W), pDZ5 F303W (*HtrA2* S306A F303W), pDZ10 V325D (*HtrA2* V325D), pDZ5 V325D (*HtrA2* S306A V325D), pDZ10 I329N (*HtrA2* I329N), pDZ5 I329N (*HtrA2* S306A I329N), pDZ10 F331Y (*HtrA2* F331Y), pDZ5 F331Y (*HtrA2* S306A F331Y), pDZ5 F331W (*HtrA2* S306A F331W), pDZ5 Y361W (*HtrA2* S306A Y361W), pDZ10 Y361W (*HtrA2* Y361W), pDZ5 V364W (*HtrA2* S306A V364W), pDZ5 L367W (*HtrA2* S306A L367W), pDZ5 L377W (*HtrA2* S306A L377W), pDZ10 V226K E429L (*HtrA2* V226K E429L), pDZ10 E429L (*HtrA2* E429L), and pDZ10 R432L (*HtrA2* R432L). The plasmids were obtained in this work as described below.

### Plasmid construction

A 975-bp fragment of human HtrA2, corresponding to amino acids 134 to 458, was amplified by PCR using the plasmid pJN1 as a template and the following primers: *HtrA2*forward 5′-GCGGCCGTCCATATGGCCGTCCCTAGCC and *HtrA2*reverse 5′-GTGCTCGAGTTCTGTGACCTCAGGGGTCAC. The *Nde*I and *Xho*I restriction endonuclease sites were incorporated into the primer sequences (underlined). The *HtrA2* cDNA fragment thus obtained was cloned into the *Nde*I and *Xho*I restriction sites of the pET24a vector (Novagen, San Diego, CA, USA). The sequence of the cloned *HtrA2* was verified by nucleotide sequencing. The obtained construct was named pDZ10. Plasmid pDZ5 was generated by site-directed mutagenesis of the pDZ10 plasmid, according to the protocol of the Quick-Change Mutagenesis Kit (Stratagene). Mutations of the *HtrA2* gene: V226K, V226W, V325D, I329N, F303W, F331Y, F331W, Y361W, V364W, L367W, L377W, E429L, and R432L were introduced by site-directed mutagenesis, with the pDZ5 and pDZ10 plasmids used as templates. The oligonucleotide primers used for mutagenesis, designed according to the Quick-Change Mutagenesis Kit protocol, are listed in Table S1. The sequences of the mutated *HtrA2* variants were verified by nucleotide sequencing. The encoded HtrA2 proteins were tagged with His_6_ at the C-terminus.

### Purification of proteins

An *E. coli* BL21(DE3) strain transformed with appropriate plasmids was used to overproduce the wild-type or mutated HtrA2(134–458) proteins, with His_6_-tags at their C-terminal ends, in the pET System (Novagen, San Diego, CA, USA), and the proteins were purified by affinity chromatography on Ni-NTA columns according to the manufacturer’s instructions (Qiagen, Germany). The purity of the mutated proteins was estimated to be more than 95 % as judged by sodium dodecyl sulfate–polyacrylamide gel electrophoresis (SDS–PAGE). The concentration of HtrA2 preparations was estimated by staining with Amido Black as described previously (Lipińska et al. 1990) and by the Bradford method (Bradford 1976).

### CD measurements

The far–UV spectra (200–260 nm) of HtrA2 proteins at a concentration of 0.5 mg/ml were recorded in a 20-mM Tris–HCl pH 8.0, 100-mM NaCl, and 1-mM EDTA buffer at a temperature range of 20–85 °C (every 0.5 °C), in 1-mm path-length cells, using a JASCO J-815 (Japan) spectropolarimeter. The mean residue ellipticity was calculated according to Kelly et al. (2005) using the following equation:

1 $$ \left( \Theta \right){\text{mrw}},\lambda = {{{{\text{MRW}} \times {\Theta_{\lambda }}}} \left/ {{10 \times d \times c}} \right.} $$

where Θ*λ* is the observed ellipticity (in degrees) at wavelength *λ*, *d* is the path length (in centimeters), *c* is the concentration (in grams per milliliter), ${\text{MRW}} = {{M} \left/ {{\left( {N - {1}} \right)}} \right.}$, *M* is the molecular mass of the polypeptide chain (in Daltons), and *N* is the number of amino acids in the chain; the number of peptide bonds is *N* − 1. To assay melting point temperatures (*T*_m_) of HtrA2 variants, far-UV CD signals recorded at 207 nm, at 20–85 °C (every 0.5 °C), were used. The *T*_m_ values were calculated by fitting the ellipticity data to the sigmoidal Boltzmann curve using the program OriginPro 8.6.

### Fluorescence quenching measurements

The fluorescence quenching measurements were carried out using a Perkin Elmer LS55 luminescence spectrometer connected to a Julabo F12 water bath. The protein samples were prepared and measured in 20 mM Tris–HCl pH 8, 100 mM NaCl, and 1 mM EDTA buffer at a temperature range of 20–45 °C (every 5 °C) in 1-cm path-length cuvette. Temperature was constantly controlled throughout the experiment by placing the thermocouple directly into the cuvette holder. The quenching experiments were carried out by the addition of a small aliquot of acrylamide stock solution (6 M) to the protein solution (0.4 − 1.7 μM). The spectra were recorded at the range of 308–400 nm, and the excitation wavelength was 295 nm. Ten-nanometer bandpasses were used for both excitation and emission. Fluorescence values were corrected for dilution effects, residual emission, Raman scattering, and absorption of light by acrylamide. The molar extinction coefficient (*ε*) of acrylamide at 295 nm is 0.25 M^−1^ cm^−1^ (Eftink and Ghiron 1981). Since both types of quenching, dynamic and static, may contribute to the overall quenching effect of acrylamide on the Trp fluorescence, data were analyzed according to the modified Stern–Volmer equation:

2 $$ \frac{F}{{{F_0}}} = \sum\limits_i {\frac{{{f_i}}}{{\left( {1 + {K_{\text{svi}}}\left[ Q \right]} \right)\exp {V_i}\left[ Q \right]}}} $$

where *F*_0_ is the fluorescence intensity in the absence of quencher, *F* is the fluorescence intensity in the presence of a quencher, *K*_svi_ is a dynamic quenching constant, *V*_*i*_ is a static quenching constant, and *f*_*i*_ is the fraction contribution of component *i* at experimental excitation and emission wavelengths. The average bimolecular rate quenching constant (*k*_*q*_) was calculated from *k*_*q*_ *= K*_svi_ */τ*_0_, where *τ*_0_ is the mean fluorescence lifetime in the absence of a quencher.

The Stern–Volmer equation was fitted to the experimental data by an iterative nonlinear least square method (Stryjewski and Wasylewski 1986). The calculations were performed with the assumption that the error of single measurements is equal to 1 % of the measured value (Stryjewski and Wasylewski 1986).

### Time-resolved fluorescence measurements

Time-resolved fluorescence decay measurements were performed by a photon-counting pulsed nanosecond spectrometer. Fluorescence decays were measured using homemade apparatus based on IBH products (IBH, Glasgow, UK). It consisted of (a) an IBH Data Station Hub (configurable control hub for photon counting and timing instruments); (b) a picosecond photon detection module, model TBX-04; and (c) a NanoLED pulsed diode light source, model N-295. All measurements were performed at temperature range of 20–45 °C (every 5 °C) in a buffer containing 20 mM Tris–HCl pH 8.0, 100 mM NaCl, and 1 mM EDTA. The excitation wavelength for tryptophan residues was 295 nm. Fluorescence decays were observed using a cutoff filter >320 nm. Before measurements, all samples were centrifuged (25,000×*g*, 10 min) to remove all undissolved impurities. The concentrations of proteins were in the range of 4–6 μM. Ludox (colloidal silica) in water was used as a reference (*τ* = 0.00 ns). The error of the calculated values of the fluorescence parameters was assumed to be 5 %. Intensity decay data were analyzed using the following multi-exponential decay law:

3 $$ I(t) = \sum\limits_{{i = 1}}^n {{\alpha_i}\exp \left( {{{{ - t}} \left/ {{{\tau_i}}} \right.}} \right)} $$

where *α*_*i*_ and *τ*_*i*_ are the pre-exponential factor and decay time of component *i*, respectively. The fractional fluorescence intensity of each component is defined as ${\tau_0} = {{{\sum\limits_i {{\alpha_i}\tau_i^2} }} \left/ {{\sum\limits_i {{\alpha_i}{\tau_i}} }} \right.}$. The software used for analysis was from IBH. The goodness of the fit was verified by residuals distribution and minimization of the reduced *χ* ^2^ test value.

### Dynamic light scattering

The dynamic light scattering measurements were performed using the DynaPro-MS800 instrument (Protein Solutions Inc., Charlottesville, VA, USA). This instrument monitors the scattered light at 90°. His-tagged HtrA2S306A (1 mg/ml) in 20 mM Tris–HCl pH 8, 100 mM NaCl, and 1 mM EDTA buffer was centrifuged (25,000×*g*, 10 min, 4 °C) and was loaded into a 45-μl quartz cuvette. Measurements were performed in the temperature range of 20–45 °C (every 5 °C) after an equilibration time of 5 min at a given temperature. At least 30–40 measurements each of 12 s duration were collected. The refractive index and viscosity values were taken for the water as provided by the software. The translational diffusion coefficient (*D*_T_) of the protein was calculated from the autocorrelation of scattered light intensity. In our case, the intensity profile showed the presence of only one species.

The hydrodynamic radius (*R*_H_) was derived from *D*_T_ using the Stokes–Einstein equation:

4 $$ {R_{\text{H}}} = {{{{k_{\text{B}}}T}} \left/ {{6\pi \eta {D_{\text{T}}}}} \right.} $$

where *k*_B_ is the Boltzman constant, *T* is temperature in Kelvin, and *η* is the solvent viscosity. This equation considers the particles as rigid spheres with a diameter related to the translational diffusion coefficient, which in this instance depends on the size and conformation of the particle at a given temperature and solvent viscosity. Data sets obtained were analyzed using the Dynamics software, provided by the supplier.

### Analysis of the protease activity

The HtrA2 protease activity using β-casein as a substrate was analyzed at the temperature range of 20–45 °C (every 5 °C) as described by Skórko-Glonek et al. (1995). The wild-type (wt) HtrA2 (0.07 μM) was incubated with β-casein (15 μM) in 50 mM Tris–HCl pH 8.0, 10 % glycerol buffer, in the final volume of 100 μl (the final HtrA2 monomer/β-casein molar ratio was 1: 214). Samples were withdrawn every 5 min, for 45 min. The reaction was terminated by addition of Laemmli lysis buffer and immediate freezing at −20 °C. The samples were then resolved by 12.5 % SDS–PAGE and gels were stained with Coomassie Brilliant Blue. The electrophoregrams were analyzed densitometrically using the 1DScan EX (Scanalytics Inc.). HtrA2 proteolytic activity with fluorescent substrate Ala(Mca)IRRVSYSF-ANB-NH2 was assayed as described by Martins et al. (2003). The reaction was carried out in the presence of 0.1 μM HtrA2, 7.5 μM substrate peptide, in 50 mM Tris–HCl pH 8.0, 1 mM DTT, and 0.5 mM EDTA buffer at temperatures 20–45 °C (every 5 °C) for 40 min, and fluorescence was measured as a function of time, using a Perkin Elmer LS55 luminescence spectrometer connected to a Julabo F12 water bath, with excitation at 315 nm and emission at 400 nm. The linear region of the fluorescence versus time plot was used to calculate reaction rate. The activity was calculated using a standard curve prepared with Fmoc-β-(7-methoxy-coumarin-4-yl)-Ala-OH at temperatures 20–45 °C (every 5 °C). In kinetic measurements, the fluorescent substrate was used at concentrations ranging from 0.025 to 15 μM and the enzyme at concentrations in the range 10–100 nM, and fluorescence was measured at 30 °C as a function of time, using a Fluorostar Omega microplate reader (BMG, Germany). At least five measurements were performed and the standard deviation did not exceed 10 %. Steady-state kinetic parameters were obtained by fitting data to the Boltzmann form of the Michaelis–Menten equation $\left( {y = \frac{{{{A}_{1}} - {{A}_{2}}}}{{1 + {{e}^{{\left( {x - x0} \right)/{\text{d}}x}}}}} + {{A}_{2}}} \right)$, where *A*_1_ and *A*_2_ were the maximal and minimal reaction rates, respectively, and *x* was the substrate concentration. The non-linear least squares subroutine in the program OriginPro 8.6 was used for the fitting. To test the influence of a peptide on HtrA2 activity, Acetyl-GQYYFV-COOH (Martins et al. 2003) was added to 10–100 μM concentration to the reaction mixture containing 1.5 μM fluorescent substrate in the 50 mM Tris–HCl pH 8.0 and 5 % glycerol buffer, then HtrA2 was added to 20 nM concentration, and the reaction was carried out at the indicated temperature. Fluorescence was measured as a function of time, using a Fluorostar Omega microplate reader (BMG, Germany).

## Results

### Effect of temperature and peptides on HtrA2 activity

**Fig. 1:**
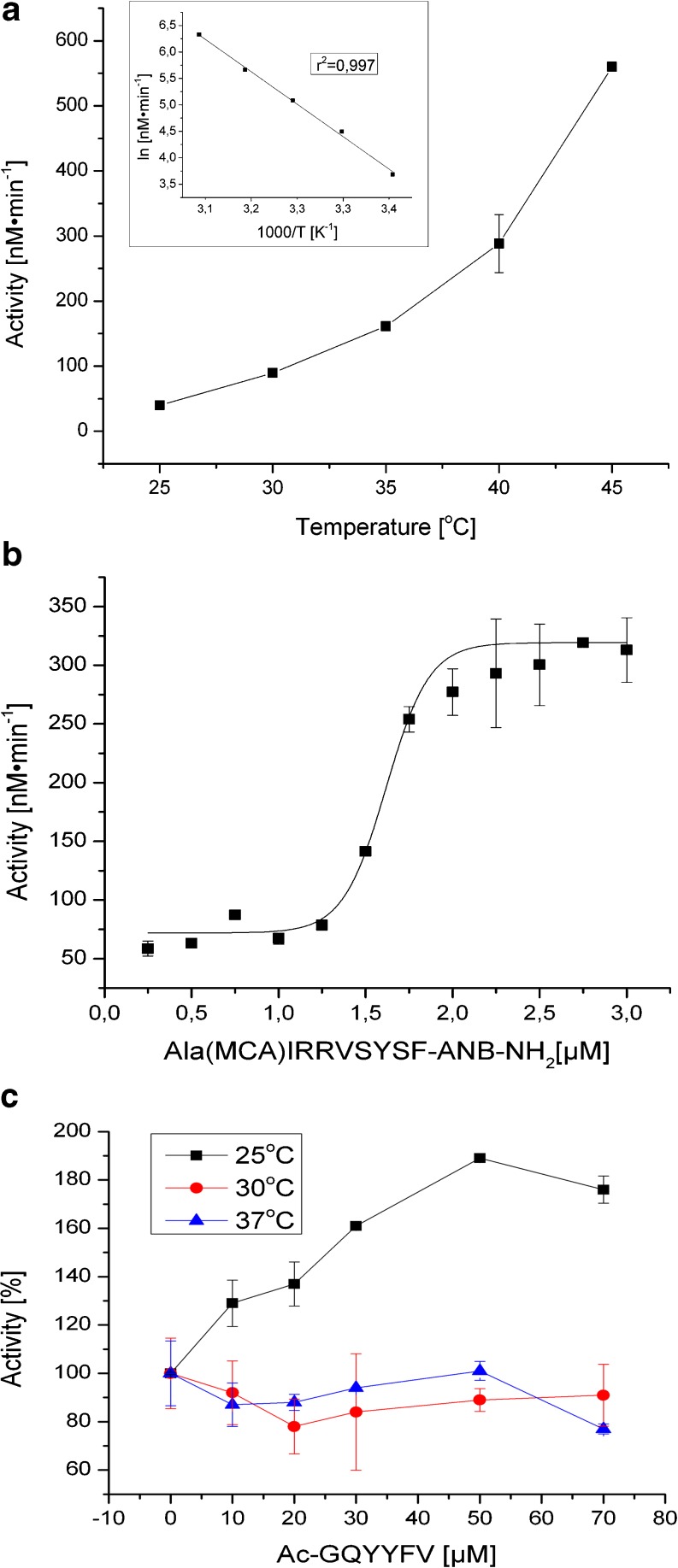
HtrA2 activity as a function of temperature and substrate concentration. Proteolytic activity was assayed using fluorescent substrate Ala(Mca)IRRVSYSF-ANB-NH2 and 0.1 μM (monomer) enzyme. **a** Temperature dependence of HtrA2 activity, assayed with saturating amount of substrate (10 μM). The *inset* presents an Arrhenius plot of the same data. The data were fitted by linear regression using OriginPro 8.6 software. The *r* ^2^ coefficient is indicated in the figure. **b** Concentration dependence of the rate of substrate cleavage by HtrA2 at 30 °C. The data were fitted to the Boltzmann form of the Michaelis–Menten equation using OriginPro 8.6 software. **c** Influence of the peptide Acetyl-GQYYFV-COOH on HtrA2 activity was measured at temperatures indicated in the graph, with 10 μM substrate. HtrA2 activity without the peptide was set as 100 %. *Error bars* are averages ± SD (*n* = 3)

**Fig. 2:**
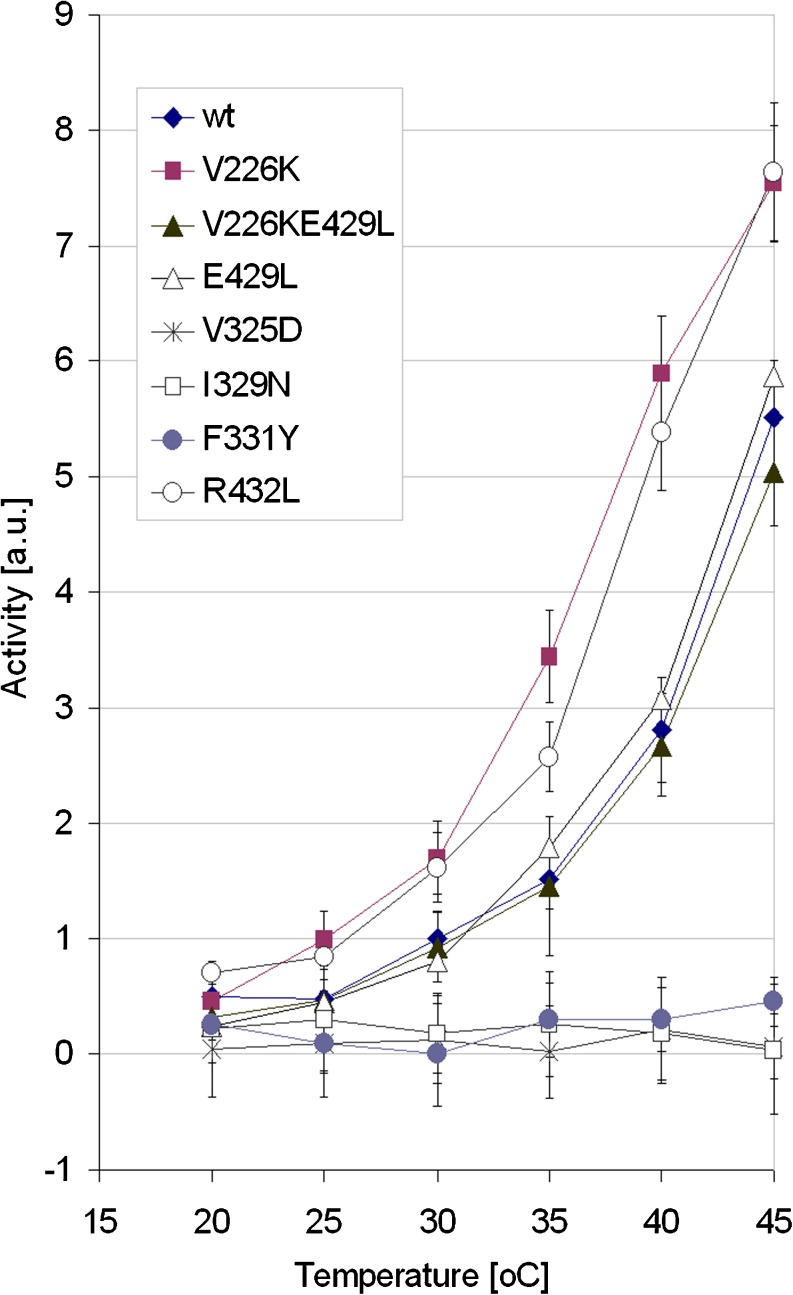
Temperature-dependent proteolytic activity of HtrA2 wt protein and HtrA2 variants. The assay of β-casein degradation was performed as described in “Materials and methods” section. Activity of the wt HtrA2 at 30 °C was arbitrarily set as 1

Our main goal was to monitor structural changes in HtrA2 during increases in temperature. In order to establish optimal conditions for structural studies, we measured the kinetics of HtrA2 proteolytic activity as a function of temperature, substrate concentration, and regulatory peptide concentration. We used two peptides: (1) a fluorescent substrate, Ala(Mca)IRRVSYSFANB-NH_2_, which is a modification of the Mca-IRRVSYSF(Dnp)KK, the HtrA2 substrate described by Martins et al. (2003), and (2) an activating peptide, Acetyl-GQYYFV-COOH (Martins et al. 2003). We found that HtrA2 activity was highly dependent on temperature: it was practically negligible at 20 °C and increased about 14-fold between 25 and 45 °C (Fig. 1a). A similar result (11-fold increase) was obtained when β-casein as a substrate was used (Fig. 2). The marked increase of HtrA2 activity in conjunction with rising temperatures is consistent with its main physiological role, which is to serve as a protein control guard and maintain mitochondrial homeostasis (reviewed in Żurawa-Janicka et al. 2010; de Castro et al. 2011). Since the use of a fluorogenic peptide permitted a more accurate quantitative assay compared to the assay with β-casein, this method was chosen for the kinetic measurements. The fluorescent substrate cleavage followed sigmoidal kinetics, characteristic for allosteric enzymes, with *K*_m_ = 1.58 ± 0.02 μM, *k*_cat_ = 0.05 s^−1^, and *k*_cat_/*K*_m_ = 31,329 M^−1^ s^−1^ (Fig. 1b). The kinetic values differ from the ones obtained by Martins et al. (2003) (*K*_m_ above 100 μM, *k*_cat_/*K*_m_ = 1,250 M^−1^ s^−1^), which may be due to the structural differences between the substrates (the internal sequences of both substrates are identical, but the flanking sequences, including the fluorescent probe/quencher pairs are different) and also to differences in assay conditions.

Previously, several peptides binding to the HtrA2 PDZ domain and activating the protease have been described (Martins et al. 2003; Gupta et al. 2004). To compare the effect of temperature and activating peptides, we used the hexapeptide Acetyl-GQYYFV-COOH (p6), with amino acid sequence identical to the biotin-GQYYFV-COOH peptide, characterized by Martins et al. (2003). We found its activating effect at 25 °C but not at 30 or 37 °C (Fig. 1c), which is in general agreement with the results obtained by Martins et al. (2003) who stated that the heat-shock-treated HtrA2 could not be further stimulated by activating peptide.

### Construction of single-Trp mutants of HtrA2

**Fig. 3:**
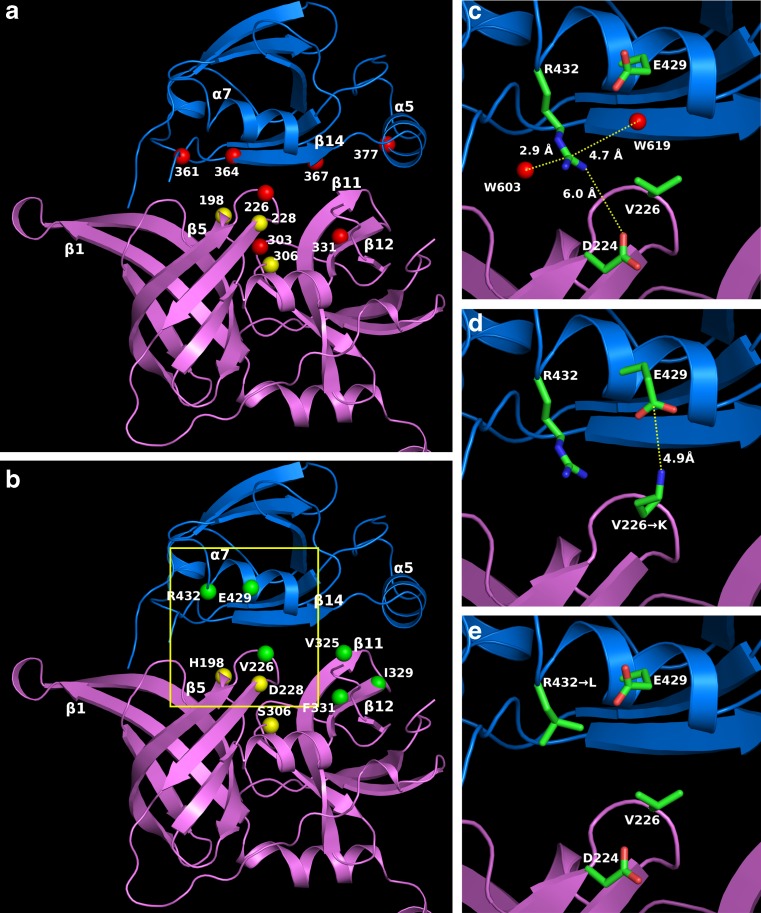
Location of amino acid substitutions in HtrA2 protease (*magenta*) and PDZ (*dark blue*) domains. Single-Trp substitutions are shown as *red balls* (**a**), other substitutions as *green balls* (**b**); the catalytic triad is indicated by *yellow balls*. The region marked with *rectangle* in **b** has been enlarged and shown in **c** (wild type), **d** (V226K variant), and **e** (R432L variant). The putative salt bridges in **c** and **d** are *marked*. The structural water molecules, W603 and W619, are shown in **c**. Figure was drawn using the PyMol program (www.pymol.org) and the HtrA2 inactive structure (PDB ID: 1LCY)

To monitor structural changes in the HtrA2 protease, we took advantage of the spectral properties of tryptophan, which has been shown to be a useful probe for studying protein structure and dynamic. Since the wt HtrA2 does not contain any Trp residue, we generated a set of single-Trp HtrA2 variants, each possessing a lone Trp residue within either the protease domain (V226W, F303W, F331W) or the PDZ domain (Y361W, V364W, L367W, L377W). We chose the residues which, according to the crystal structure of the inactive protease (Li et al. 2002), were located at the interface between the domains. The Trp residues were introduced in place of aromatic residues (Phe, Tyr) or large hydrophobic residues (Leu or Val) in order to minimize the effects of mutations on the overall protein structure and stability. The positions of the introduced mutations are shown in Fig. 3a. To avoid autodegradation, which occurs during the wt HtrA2 preparation and subsequently interferes with structural studies, we used the *HtrA2*S306A gene, encoding the proteolytically inactive HtrA2 variant, as a template for mutagenesis. Secondary structure and thermal stability of the mutated proteins were checked by CD analyses (described below). In parallel, we constructed V226W and Y361W variants without the S306A substitution and assayed their proteolytic activity. Since their activity did not differ from that of the wt HtrA2 protein (not shown), we assumed that in these cases the single-Trp substitutions did not significantly change the HtrA2 secondary and tertiary structure.

### Fluorescence-monitored temperature-dependent structural changes of HtrA2

To gain information about the possible alterations within the HtrA2 structure during activation, we examined the effect of temperature shift on the intrinsic fluorescence emission properties of the single-tryptophan HtrA2 mutants. A measure of the polarity of the immediate surrounding of a Trp residue in the protein is the position of the maximum of the emission wavelength (*λ*_em_max). Solvent studies indicate that a contact of an indole or Trp residue with a more polar and solvent-exposed environment causes a red shift of the emission maximum (Cree 1984). Table S2 lists the values of the *λ*_em_max of the corrected fluorescence emission spectra of the single-Trp HtrA2 mutants. For all Trp residues except W364, these values range from 339 to 358 nm at 20 °C (Table S2 and Fig. S1), which is indicative of a rather polar environment of the residues. The *λ*_em_max observed for a model compound *N*-acetyl tryptophanamide (NATA) of 361 nm represents a value typical for a completely solvent-exposed indole group (Lakowicz 2006). The Trp residues located at the positions 226 and 303 of the proteolytic domain and at the positions 361 and 377 of the PDZ domain possessed the most red-shifted fluorescence emission maxima (*λ*_em_max = 356.0, 358.0, 348.5, and 351.5, respectively, at 20 °C). The Trp residue with the highest fluorescence energy was W364 of the PDZ domain (*λ*_em_max = 324.7 nm at 20 °C). This residue, according to crystal structure, is deeply buried and surrounded by hydrophobic residues. The *λ*_em_max values of the studied Trps underwent slight changes upon temperature shift (20 → 45 °C) (Table S2 and Fig. S1). In the PDZ domain, the Trp residues 361 and 367 exhibited a red wavelength shift of 3 and 2.25 nm, respectively. The *λ*_em_max of W364 was also red-shifted, but only at temperatures up to 30 °C (324.7 → 326.25 nm). Conversely, at temperatures 30–45 °C, the *λ*_em_max showed a blue shift (326.25 → 323.33 nm). For NATA, the *λ*_em_max was independent of temperature. Thus, the observed wavelength shifts must be indicative of temperature-induced conformational changes in the vicinity of the Trp residues. In the case of W377, small fluctuations without a defined tendency were observed. There were no significant *λ*_em_max changes in W226, W303, and W331 of the protease domain. The lack of any defined *λ*_em_max changes in the case of W226, W303, and W377 could result from the fact that the Trp residues were located in a highly polar environment, which is indicated by very high values of *λ*_em_max at 20 °C. Analysis of HtrA2 crystal structure (PDB ID: 1LCY) indicated that in the case of W303, the polar environment could be caused by solvatation of the Trp residues by H_2_O molecules penetrating inside the structure (there are 26 H_2_O molecules at 9 Å radius) and by Asn181 residue (at a distance less than 5 Å). The high value of *λ*_em_max noted for W226 could be caused by the neighboring (at a distance less than 5 Å) polar groups of Asp224, Arg337, Glu425, and Arg432; in the case of W377, the polar environment could be provided by Arg380, Thr326, and 3 H_2_O molecules. F331W had a lower *λ*_em_max compared to W226, W303, and W377, indicating less polar environment. Thus, the lack of *λ*_em_max changes of F331W suggested that temperature did not induce any significant structural changes in the vicinity of this residue.

**Fig. 4:**
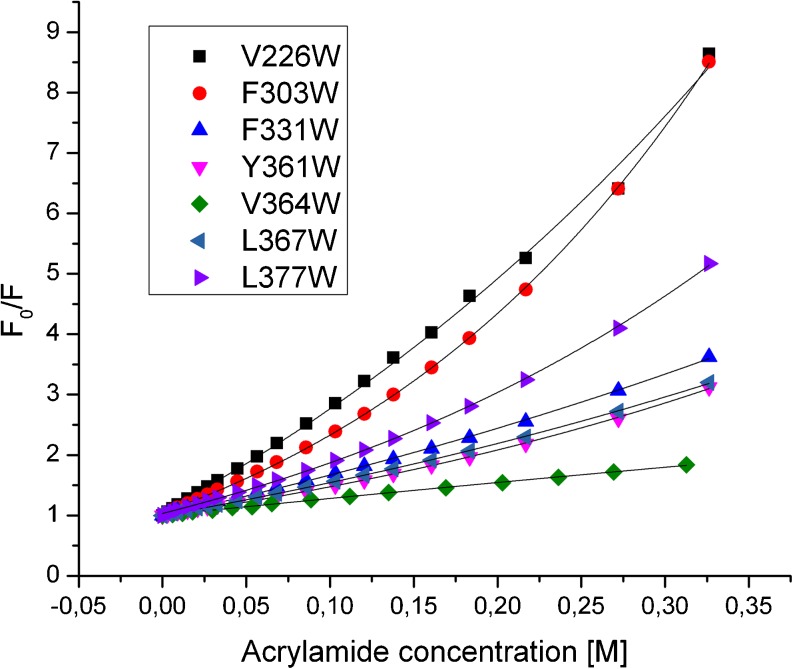
Typical Stern–Volmer quenching plots of the HtrA2 Trp mutant proteins. Tryptophan fluorescence quenching with acrylamide at increasing concentrations was measured at 30 °C. Areas under the curves representing the emission spectra at the range of 20 nm around fluorescence maximum were integrated, *F*_0_/*F* values were calculated and plotted against acrylamide concentrations. The data were fitted to Stern–Volmer equation (Eq. 2) using OriginPro 8.6 software

To expand our knowledge of the accessibility and degree of exposure of Trp residues, we performed steady-state fluorescence quenching experiments at temperatures 20–45 °C. As a quencher, we used acrylamide, a non-polar molecule known to penetrate into a protein (Mátyus et al. 2006). Acrylamide is normally expected to quench both the “exposed” and “buried” tryptophan residues primarily via a collisional mechanism (Calhoun et al. 1986). The quenching data were plotted according to the Stern–Volmer equation. The typical Stern–Volmer quenching plots for the titration of the single-Trp HtrA2 mutants at 30 °C are shown in Fig. 4. The quenching plots in the case of W331, W361, W364, and W367 were nearly linear over a wide range of acrylamide concentrations and temperatures. At high acrylamide concentration, a small upward curvature was observed in the case of W377 while a considerable non-linear increase of quenching was found for W303 and W226, suggesting the occurrence of static quenching or rather a pseudo-effect of static quenching by a dynamic quencher—acrylamide. In these cases, a static constant, *V*, has been calculated (Table 1). At some temperatures, the Stern–Volmer quenching plots did not deviate enough from linearity to assign the static constant *V*.

**Table 1:** The values of Stern–Volmer (*K*_sv_) and bimolecular quenching (*k*_*q*_) constants of the single-Trp HtrA2 protein variants

Temperature (°C)	V226W				F303W				F331W			Y361W			V364W			L367W			L377W			
	*K*_SV_ (M^−1^)	*V* (M^−1^)	*k*_*q*_ (M ns)^−1^	Class	*K*_SV_ (M^−1^)	*V* (M^−1^)	*k*_*q*_ (M ns)^−1^	Class	*K*_SV_ (M^−1^)	*k*_*q*_ (M ns)^−1^	Class	*K*_SV_ (M^−1^)	*k*_*q*_ (M ns)^−1^	Class	*K*_SV_ (M^−1^)	*k*_*q*_ (M ns)^−1^	Class	*K*_SV_ (M^−1^)	*k*_*q*_ (M ns)^−1^	Class	*K*_SV_ (M^−1^)	*V* (M^−1^)	*k*_*q*_ (M ns)^−1^	Class
20	11.22 ± 0.82	1.61 ± 0.45	1.56 ± 0.14	2	8.17 ± 0.17	2.21 ± 0.33	1.01 ± 0.03	1	6.02 ± 0.24	1.24 ± 0.06	1	3.52 ± 0.32	0.68 ± 0.08	1	2.1 ± 0.28	0.67 ± 0.13	1	4.17 ± 0.08	1.06 ± 0.08	1	6.92 ± 0.77	1.25 ± 0.21	1.04 ± 0.13	1
25	12.5 ± 0.86	1.58 ± 0.6	1.82 ± 0.15	2	9.1 ± 0.01	2.53 ± 0.55	1.17 ± 0.01	1	6.04 ± 0.12	1.29 ± 0.06	1	4.26 ± 0.16	0.85 ± 0.04	1	2.47 ± 0.27	0.82 ± 0.14	1	4.16 ± 0.57	1.11 ± 0.21	1	7.03 ± 0.49	1.52 ± 0.03	1.09 ± 0.1	1
30	13.2 ± 0.59	2.41 ± 0.75	1.99 ± 0.11	2	9.38 ± 0.09	2.4 ± 0.3	1.25 ± 0.02	1	6.23 ± 0.25	1.34 ± 0.14	1	5.29 ± 0.08	1.08 ± 0.03	1	3.37 ± 0.36	1.16 ± 0.15	1	4.39 ± 0.25	1.2 ± 0.08	1	7.38 ± 1	1.59 ± 0.25	1.19 ± 0.17	1
35	13.06 ± 0.61	3.99 ± 0.64	2.15 ± 0.16	2	9.45 ± 0.15	2.23 ± 0.23	1.32 ± 0.05	1	6.39 ± 0.12	1.40 ± 0.16	1	4.63 ± 0.01	0.96 ± 0.01	1	3.35 ± 0.39	1.23 ± 0.23	1	4.43 ± 0.43	1.27 ± 0.18	1	7.85 ± 0.35	1.78 ± 0.09	1.3 ± 0.08	1
40	14.58 ± 0.87	2.18 ± 0.89	2.58 ± 0.17	2	11.51 ± 0.1	2.24 ± 0.2	1.67 ± 0.03	2	6.47 ± 0.68	1.46 ± 0.44	1	4.57 ± 0.18	0.96 ± 0.05	1	3.12 ± 0.23	1.19 ± 0.11	1	4.38 ± 0.3	1.28 ± 0.11	1	8.31 ± 0.91	1.77 ± 0.29	1.43 ± 0.17	1
45	12.55 ± 0.78	3.23 ± 0.07	2.48 ± 0.25	2	11.78 ± 0.97	2.72 ± 0.21	1.81 ± 0.18	2	6.40 ± 0.05	1.55 ± 0.14	2	4.56 ± 0.42	0.97 ± 0.1	1	3.4 ± 0.27	1.39 ± 0.23	1	5.08 ± 0.52	1.55 ± 0.22	2	8.3 ± 0.53	2.14 ± 0.39	1.49 ± 0.12	1

Quenching of Trp fluorescence by acrylamide at increasing temperatures was measured for each HtrA2 variant, and the dynamic quenching constants, *K*_sv,_, were calculated. For some variants, a static quenching constant, *V*, has also been calculated, as explained in the text. Using the *K*_sv_ values and the values of the mean fluorescence lifetimes of the Trp residues (τ_0_), presented in Table S2, the average bimolecular quenching constants, *k*_*q*_, were derived. All calculations were done as described in the “Materials and methods” section. Basing on the *k*_*q*_ values, the Trp residues were classified as: 1—moderately exposed to the solvent [0.6 < *k*_*q*_ < 1.5] or 2—exposed to the solvent [1.5 < *k*_*q*_], according to the classification of the degree of exposure proposed by Merrill et al. (1993) and Calhoun et al. (1986)

The Stern–Volmer plots showing upward curving were analyzed in terms of the static and dynamic quenching constants. The static constant ranged from 3.99 for W226 to 1.25 for W367 (Table 1). It seems that the apparent static component is due to the quenchers being adjacent to the fluorophore at the moment of excitation (“sphere of action”) (Lakowicz 2006). These closely spaced fluorophore–quencher pairs are immediately quenched and thus appear to be a dark complex. With the rise of quencher concentration, the probability increases that a quencher is within the first solvent shell of the fluorophore at the moment of excitation. Such an effect is best visible for the residues highly exposed to the solvent (and to the quencher).

The Stern–Volmer quenching constants (*K*_sv_) of the Trp residues ranged from 2.1 M^−1^ for W364 to 11.22 M^−1^ for W226 at 20 °C (Table 1). To compare directly the accessibility of the fluorophores to the quencher, the bimolecular rate constant, *k*_*q*_, was calculated (*k*_*q*_ *= K*_sv_ */τ)*. The mean fluorescence lifetimes (*τ*_0_) were used for the calculation of the *k*_*q*_ values. The results are shown in Table 1. In general, the Trp residues with the emission maxima (*λ*_em_max) at longer wavelengths had higher values of *k*_*q*_ compared to the one (W364) with the shortest *λ*_em_max, especially at 20 °C (Table 1 and Fig. S1). The relatively high values of both *k*_*q*_ and *λ*_em_max were found for W226, W331, W367, W377, and W303, the lowest—for W364. However, the W361 had a low *k*_*q*_ and a high *λ*_em_max. The low *k*_*q*_ value could result from the relatively high fluorescence lifetime. In water at 20 °C, the *k*_*q*_ of the acrylamide quenching of indole has been repeatedly found in the range of 6.5 × 10^9^–7.5 × 10^9^ M^−1^ s^−1^, which is approximately equal to the theoretical value of 7.4 × 10^9^ M^−1^ s^−1^ (Calhoun et al. 1986). The values calculated for all HtrA2 variants were significantly lower. Reduction of *k*_*q*_ can result from steric shielding of the fluorophore, but the bimolecular quenching constant depends not only on the depth of the fluorophore in the macromolecule but also on the rate of the rotational diffusion. Faster rotational diffusion provides more opportunities for quenching and higher values of *k*_*q*_. HtrA2 exists as trimer with molecular weight of 108.5 kDa, and this might explain the low values of *k*_*q*_.

**Fig. 5:**
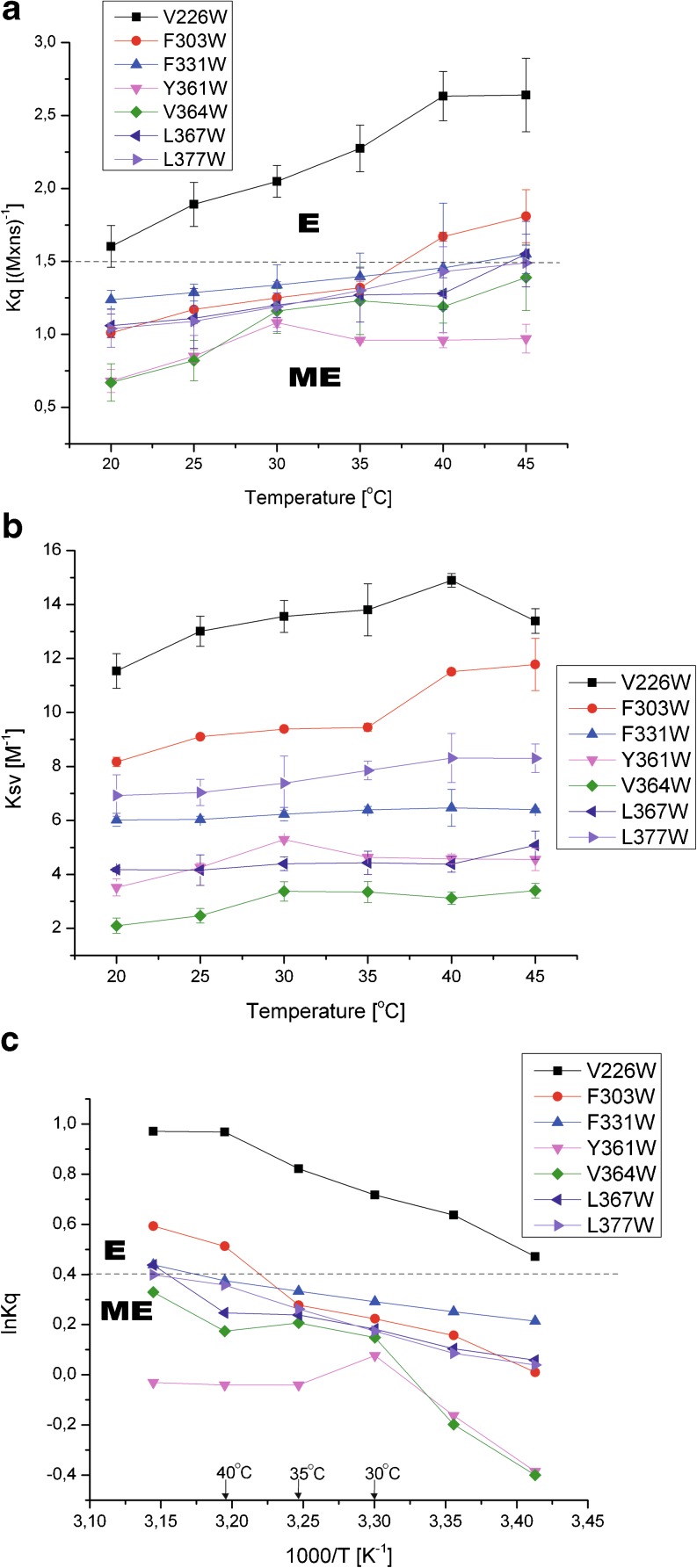
Quenching of fluorescence of the tryptophan side chains of HtrA2 variants with single Trp residues by acrylamide. **a** The bimolecular quenching constants (*k*_*q*_) values of HtrA2 variants as a function of temperature; the data correspond to means ± SD of three or two different experiments. **b** The Stern–Volmer quenching constants (*K*_sv_) values of HtrA2 protein variants as a function of temperature; the data correspond to means ± SD of three or two different experiments. **c** Arrhenius plots for the acrylamide quenching of HtrA2 variants—the data as in **a**. The *dashed line* classifies the Trp residues into two categories: *ME* moderately exposed; *E* exposed

On the basis of *k*_*q*_ values the individual Trp residues in HtrA variants were placed into two categories, according to the grouping suggested by Calhoun et al. (1986) and Merrill et al. (1993). These categories include moderately exposed and exposed Trp residues in proteins (Table 1 and Fig. 5a, c). All Trp residues became more exposed as temperatures changed from 20 to 45 °C, but the patterns of transition varied. In the protease domain, W303 underwent a transition from a moderately exposed to an exposed Trp (*k*_*q*_: 1.01 → 1.81 M^−1^ ns^−1^)—the transition was almost linear up to 35 °C and then its rate increased; the exposed state was reached between 35 and 40 °C. In the W226, the Trp was in an exposed state and *k*_*q*_ values increased up to 40 °C (*k*_*q*_: 1.56 → 2.48 M^−1^ ns^−1^). W331 showed a very minor, almost linear transition from the moderately exposed to the exposed state at 45 °C (*k*_*q*_: 1.24 → 1.55 M^−1^ ns^−1^). All Trp residues of the PDZ domain were initially in the moderately exposed status and the exposure of W364, W367, and W377 increased, reaching or just crossing the boundary between the moderately exposed and the exposed state. It should be noted that the rate at which *k*_*q*_ for W364 increased slowed down above 30 °C. In the case of W361, the Trp exposure increased up to 30 °C and then decreased. It can be seen in Table 1 and Fig. 5a that as temperatures changed, the most exposed status was reached by the Trp residues W226 and W303 of the protease domain. Their exposed status is consistent with the fact that they are located in the loops close to the active site D228 and S306, respectively, and these residues should be accessible to a solvent and substrate in the active enzyme.

Taking into consideration the fact that several Trp residues (W226, W303, W361, W377) had long mean fluorescence lifetimes, which had an impact on the *k*_*q*_ values, we also presented the Stern–Volmer quenching constants (*K*_sv_) along with the bimolecular rate constants (Fig. 5b). It can be seen that the changes of the *K*_sv_ values with temperature are similar to those observed for the *k*_*q*_ values, especially the discontinuity of the W303 plot at 35 °C and of the W361 and W364 plots at 30 °C. Presumably, at these transition temperatures, conformational changes occur in the HtrA2 domains, in the PDZ domain at 30 °C and in protease domain—at 35 °C. These discontinuities, pointing to conformational changes, are even more pronounced in Arrhenius plots (Fig. 5c) which essentially represent the temperature effect on the overall reaction rate. In addition to the downward (W364 and W361) or upward (W303) discontinuities, another can be observed for W226 of protease domain at 40 °C. In conclusion, the conformational changes occur in the microenvironment of all the Trp residues, but they display different temperature dependence.

### Residues important for HtrA2 activity

To support our conclusions that structural changes occur at the interface between the protease and PDZ domains when HtrA2 activity increases, we introduced mutations aimed at changing the interdomain interactions. We generated HtrA2 variants in which, in the protease domain, several hydrophobic amino acids were substituted with the polar ones: V226K, V325D, I329N, and F331Y, and in the PDZ domain, a polar residue was exchanged for a hydrophobic one (R432L). Similarly as in the case of the Trp mutants, we chose the residues which, according to the crystal structure of the inactive protease (Li et al. 2002), were located at the interface between the domains. We expected that changing interaction between the domains should influence the opening of the structure and activity during temperature increase. The positions of the introduced mutations are shown in Fig. 3b. V226 is located in the loop between strands β5 and β6 and loosely occupies the peptide-binding groove of the PDZ domain. V325 is placed in strand β11, I329 and F331—in strand β12, and they belong to the region of intramolecular contact, interacting with the hydrophobic residues of the β14 and α5 strands of PDZ domain. R432 is located in a loop between α7 and β17 (Li et al. 2002). We also constructed a set of HtrA2 variants carrying, in addition to the described mutations, the S306A substitution. The latter, whose preparations were free of contamination with degraded HtrA2, served for the CD analyses (described below) aimed at showing that the substitutions did not significantly affect protein secondary structure and thermal stability.

Proteolytic activity of the protease variants as a function of temperature was assayed (Fig. 2). The activity of the V226K variant increased significantly compared to that of the wt HtrA2. A possible reason was that in V226K, a salt bridge between Lys226 and Glu429 of the PDZ domain might be formed (Fig. 3d). According to the HtrA2 crystal structure (Li et al. 2002), the distance between the Cα 226 and Cα 429 is 9.8 Å, which should make formation of such bridge possible. To clarify this situation, we created an additional variant, HtrA2 V226K E429L, in which the salt link formation was no longer possible and found that the increase of activity observed for the V226K ceased to exist. Furthermore, activity of the control variant, E429L, was very similar to that of wt HtrA2 (Fig. 2). These results showed that abolishing hydrophobic interactions between V226 of the protease domain and PDZ domain and formation of new interactions led to an increase in HtrA2 proteolytic activity. A very similar increase in activity was found for the R432L variant (Fig. 2). The possible explanation for the increase in activity is removal of a salt link formed by R432 and D224, stabilizing interactions between the domains (Fig. 3c, e). Although the distance between the ionic centers of R432 of the PDZ domain and D224 of the protease domain is about 6 Å in the inactive structure (Fig. 3c), it is likely that an “ionic lock” contributing to the stabilization of HtrA2 wt in the inactive form could be water-mediated. In a recent work (Sabarinathan et al. 2011), it is convincingly proven that water-mediated ionic interactions are quite common in protein structures and the typical distance between the interacting centers is close to just 6 Å. A careful inspection of structural water in the X-ray structure of inactive HtrA2 wt (PDB ID: 1LCY (Li et al. 2002)) indicates that there is no H_2_O molecule between R432 and D224 but that there are two structural waters, i.e., W603 and W619 flanking the R432 guanidyl at opposite sides at the H-bond distances (at ca. 2.9 and 4.7 Å, respectively) (Fig. 3c). Using crystal measurement methodologies, involving liquid nitrogen temperatures for capturing and maintaining crystals, one can speculate about the freezing (in the literal and figurative sense) of both water molecules in local minima. At ambient temperature, either W603 or W619 (or both) could dynamically assume the mediating position. This seems even more likely if one notices that the potential water-mediated ionic bridge R432-H_2_O-D224 would be on the protein surface, exposed to crystal-packing effects. On activation in the R432L variant (Fig. 3c, e), this hypothetical water-mediated ionic lock could be released.

We found that the I329N, V325D, and F331Y variants were inactive (Fig. 2). These results indicate that the presence of hydrophobic residues at the positions 325, 329, and 331 of the β11 and β12 strands are important for maintaining the proper structure of the protease domain, most probably via hydrophobic interactions.

### Secondary structure analysis and thermal denaturation of HtrA2 variants

**Fig. 6:**
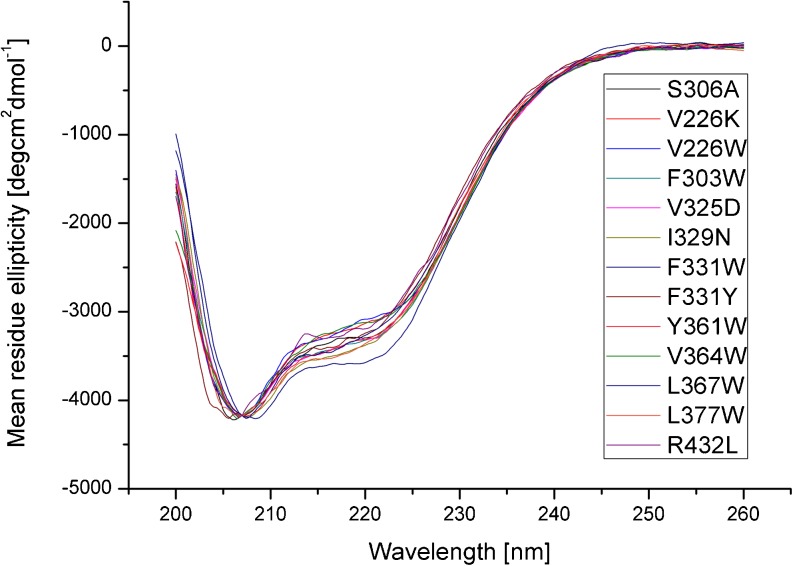
Circular dichroism analysis of HtrA2 protein variants. The far-UV spectra of HtrA2S306A protein (control) and of HtrA2S306A carrying substitutions indicated in the graph were obtained at 20 °C, and the mean residue ellipticity was calculated as described in “Materials and methods”

To ensure that the introduced mutations have not affected the overall protein secondary structure, we undertook far-UV CD spectroscopy. Changes in protein secondary structure influence the far-UV CD spectra (180–260 nm); at these wavelengths, the chromophore is the peptide bond, and the signal arises when it is located in a regular, folded environment (Kelly et al. 2005). Figure 6 shows the comparison of the CD spectrum of HtrA2S306A (control) with those of HtrA2 mutant proteins at 20 °C. It should be noted here that all the HtrA2 variants tested had the S306A substitution. All spectra have a very deep trough at about 209 nm, representing α helical structures. There are only minor variations in the region 209–220 nm. The fact that the proteolytic activity of the V226W and Y361W variants (without the S306A substitution) did not differ significantly from that of the wt HtrA2 (results not shown) is an indication that the small variations in their CD spectra do not represent significant structural changes. In general, the spectra of the HtrA2 variants and of HtrA2S306A were very similar, indicating that the mutations have not significantly modified the secondary structure of the HtrA2 mutant proteins.

HtrA2 variants were subjected to thermal denaturation conditions in order to obtain the melting temperature (*T*_m_) of HtrA2S306A, of the single-Trp mutants and of the mutants showing changes in proteolytic activity. When a protein is heated, its unfolding leads to changes in the CD spectrum (Greenfield 2006). We analyzed CD values at 207 nm, at increasing temperatures (20–85 °C). We chose the wavelength at which the temperature-dependent changes of the HtrA2S306A CD signal were the highest. The *T*_m_ value of each HtrA2 variant was calculated from its thermal denaturation curve as described in “Materials and methods” and is presented in Table S3. The values show that the Trp mutations did not significantly affect the thermal stability of HtrA2S306A. The *T*_m_ values of HtrA2S306A (control) and of V226W, F303W, F331W, Y361W, V364W, L367W, and L377W were found at 70.71 °C and in the range from 68.63 to 72.19 °C, respectively. Very similar *T*_m_ values found for HtrA2S306A and the Trp variants indicate that the mutations did not cause significant destabilizing or stabilizing effects on the thermal denaturation course of HtrA2S306A. Melting temperatures obtained for the HtrA2 variants with substitutions affecting HtrA2 activity (V226K, I329N, F331Y, and R432L) were found in the range from 67.39 to 72.47 °C, which is again very close to the *T*_m_ of HtrA2S306A and indicates that the substitutions did not significantly affect the thermal stability of these variants.

In summary, the far-UV CD analysis of the mutated proteins confirmed that their secondary structure and thermal stability were not significantly changed compared to the control HtrA2. Thus, we believe that our conclusions based on the analysis of the HtrA2 variants are valid, and the observed and discussed changes in proteolytic activity of some variants must be due to the subtle changes in the protease tertiary and, probably, quaternary, structure.

Our results regarding the HtrA2 *T*_m_ value are in general agreement with the work of Zhang and Chang (2004) who found, using far-UV CD spectroscopy, that there were no significant changes in the HtrA2 secondary structure from 25 to 70 °C, while their differential scanning calorimetry data indicated that HtrA2 exhibits a thermal transition beginning at around 61 °C.

### Dynamic light scattering

To gain more information concerning structural changes occurring in HtrA2 molecule during temperature up-shift, we measured DLS. DLS is an established technique used to measure hydrodynamic sizes, polydispersities and aggregation effects of protein samples (Berne and Pecora 2000). The DLS data (Table 2) showed that an average hydrodynamic radius of HtrA2 S306 particles increased almost linearly from 5.84 nm at 20 °C to 6.34 at 40 °C and to 6.56 nm at 45 °C. Hence, the radius increased approximately by 8 and 12 % at 40 and 45 °C, respectively. An average polydispersity at all temperatures was between 23 and 26 %, indicating that monodispersity could be assumed and that HtrA2 forms a uniformly sized oligomer rather than a non-homogeneous mixture of aggregates. Furthermore, the oligomer remained stable at all temperatures. These results support the idea that during temperature increase, the HtrA2 oligomers undergo relaxation accompanied by an increase in size. Our finding that HtrA2 oligomers are stable up to 45 °C agrees well with the size exclusion chromatography results of Zhang and Chang (2004), who showed that the elution positions of HtrA2 at 25 and 55 °C were similar.

**Table 2:** Dynamic light scattering analysis of HtrA2 protein—changes of the hydrodynamic radius with temperature

Temperature (°C)	Diffusion coefficient^a^ (10^−13^ m^2^ s^−1^)	Hydrodynamic radius^b^ (nm)	Polydispersity^c^ (%)
20	360.68 ± 12.62	5.84 ± 0.21	28 ± 4
25	405.13 ± 6.89	5.96 ± 0.11	26 ± 2
30	451.49 ± 2.38	6.06 ± 0.03	25 ± 1
35	501.67 ± 4.24	6.15 ± 0.05	24 ± 3
40	544.04 ± 0.41	6.34 ± 0.01	26 ± 1
45	584.38 ± 20.33	6.56 ± 0.23	23 ± 1

The dynamic light scattering measurements of HtrA2S306 protein were performed at the temperature range of 20–45 °C and the translational diffusion coefficients were calculated

^a^The diffusion coefficient represents the translational diffusion coefficient of the molecule in solution

^b^Mean hydrodynamic radius derived from the measured translational diffusion coefficient using the Stokes–Einstein equation

^c^Polydispersity divided by the hydrodynamic radius

## Discussion

To gain information about the possible temperature-induced alterations within the HtrA2 structure, we examined the effect of temperature shift on fluorescence properties of the Trp residues located at the interface between the protease and PDZ domains (Fig. 3a). Experiments in which fluorescence quenching by acrylamide was assayed showed that all Trp residues became more exposed to the solvent upon temperature shift, indicating that the structure relaxed (Fig. 5a, b). However, the patterns of transition varied significantly. The most pronounced temperature-induced structural changes were observed in two regions: (1) the vicinity of the active center (W303 and W226) and (2) the peptide recognition motif YIGV of the PDZ domain (W361 and W364). The Trp residues at positions 303 and 226 showed an almost linear increase of exposition to the quencher at temperatures up to 35 and 40 °C, respectively (Fig. 5a, b). Above these temperatures, discontinuities of the Arrhenius plots are visible (Fig. 5c), indicating a local structural transition within the active site. The residue W303 in particular, located close to Ser306 of the active triad, shows a significant increase of exposition to the quencher at 35–40 °C. A different kinetics of quenching was observed for W361 and W364 of the peptide recognition motif: Exposure of these residues increased almost linearly up to 30 °C, and then significant changes in exposure rates were observed (Fig. 5a–c). Presumably, at these transition temperatures, shown by discontinuities in the *k*_*q*_, *K*_sv_, and Arrhenius plots (Fig. 5a–c), significant conformational changes occurred. The kinetics of quenching correlated well with the observed red shifts of the wavelength maxima at 20–30 °C of W361 (348.5 → 350.5 nm) and W364 (324.7 → 326.25 nm).

The lack of discontinuities in the Arrhenius plots of the W377 (PDZ, α5) and W331 (protease domain, β12) residues (Fig. 5c) and of significant changes in the wavelength maxima of their fluorescence (Table S2) suggested that there were no significant structural changes in the vicinity of these residues. These residues belong to the predicted region of intramolecular contact (Li et al. 2002). This contact is probably essential for stabilization of the HtrA2 structure. Its importance was confirmed by the fact that the HtrA2 F331Y (β12) and two other HtrA2 variants, V325D (β11) and I329N (β12), carrying substitutions in the region of intramolecular contact of protease domain, were inactive (Fig. 2). Consistently, the substituted residues are highly conserved among the HtrA proteases (Li et al. 2002; Singh et al. 2011).

Taken together, the results of the Trp fluorescence analyses indicate that upon increase of temperature HtrA2 structure relaxes, the interface between the protease and PDZ domains becomes more exposed to the solvent, and significant conformational changes occur gradually at 30, 35, and possibly 40 °C—the changes involving both the PDZ and protease domains. These changes lead to a better accessibility of the active triad and the peptide recognition motif. This conclusion correlates well with proteolytic activity measurements, showing a marked and gradual increase of activity above 30 °C, continuing up to 45 °C (Fig. 2).

Relaxation of the HtrA2 structure during temperature increase is also supported by the DLS data showing that the radius of the particle increased by approximately 12 % between 20 and 45 °C (Table 2). The linear increase of the HtrA2 particle size between 20 and 40 °C is in agreement with the idea that the HtrA2 trimer relaxes/opens gradually when the temperature rises. Since both DegS and HtrA2 are trimeric and are inhibited by their PDZ domains (reviewed by Krojer et al. 2010; Clausen et al. 2011; Singh et al. 2011), we compared dimensions of the inactive and active DegS trimers (Wilken et al. 2004) and found that the increase of size was rather minor, not exceeding 1 %. Similar analysis of the inactive (PDB ID: 1KY9 (Krojer et al. 2002)) and active (PDP ID: 3CS0 (Krojer et al. 2008)) HtrA(DegP) trimers showed an increase of 7 ± 1 %. Thus, our DLS results suggest that HtrA2 structural change upon temperature shift is relatively large.

To support our conclusions that structural changes occur at the interface between the protease and PDZ domains when HtrA2 activity increases, we introduced mutations aimed at changing the interdomain interactions (Fig. 3b–e). We expected that changing interactions between the domains should influence the opening of the structure and activity during temperature increase. Indeed, substitution of V226 with Lys, diminishing hydrophobic interactions of V226 with the peptide-binding groove of PDZ domain (specifically, with the Y428 of α7), caused a significant increase of proteolytic activity (Fig. 2). Since the increase was abolished in the variant V226KE429L, lacking Glu429 of the PDZ domain, we believe that the observed up-shift in activity was caused by formation of a salt bridge between Lys226 and Glu429 (Fig. 3d). This should liberate the peptide-binding groove and thus permit substrate binding. We also found that substitution of Arg432 of the PDZ domain caused an up-shift in HtrA2 activity (Fig. 2). In the R432L variant, a salt bridge between R432 of the PDZ domain and D224 of the protease domain, possibly existing in the wt protein, would be lost, thus decreasing interdomain interactions (Fig. 3c, e). In conclusion, the above results support the model predicting that a change of interactions between the PDZ and protease domains occurs during HtrA2 activation. It has been shown previously that single-amino acid substitutions abolishing formation of salt bridges in the interface region of DegS caused an up-shift in the protease activity (Sohn et al. 2007).

Our results also suggest that not all interdomain contacts are broken upon activation, since the variants V325D, I329N, and F331Y, with impaired hydrophobic interactions between the domains, were inactive at all temperatures. As discussed earlier, these interactions are probably important for HtrA2 structural integrity. The fact that the point mutation M323R, described by Li et al. (2002), abolished HtrA2 activity also indicates the importance of the interdomain contacts in this region.

We believe that our results experimentally support and expand the model of HtrA2 activation, derived from crystal structure of the inactive form of the protease, and provide an insight into the mechanism of the temperature-induced changes that lead to activity increase. It is tempting to speculate that when temperature increases, HtrA2 structure relaxes and conformational changes are gradual, with changes in the PDZ domain occurring first, at about 30 °C, which leads to increased accessibility of the peptide binding groove, followed by increased exposure of the active site of the protease domain at above 35 °C. This is consistent with the physiological role of HtrA2, which functions in cells at temperatures close to 37 °C and whose activity is in high demand at stress conditions, including increased temperature (Gray et al. 2000). At physiological temperatures, HtrA2, according to this scenario, could bind either a substrate or a regulatory peptide to the groove in the PDZ domain, which would then stabilize the active form of the enzyme. Our finding that HtrA2 kinetics is typical for allosteric enzymes is in agreement with this hypothesis.

The proteolytic activity of HtrA from *E. coli* and HtrA from *T. maritima* (HtrA_TM_) is regulated in a temperature-dependent manner. In both cases, at elevated temperatures, the HtrA structure opens (Kim et al. 2008; Krojer et al. 2008, 2010; Sobiecka-Szkatula et al. 2009). It seems that regulation of activity by temperature, involving structural changes and the opening of the enzyme molecule, is a common strategy of bacterial and eukaryotic HtrAs.

It is not known whether the structural changes occurring during HtrA2 activation by temperature are the same as during activation by peptides; however, our finding is that HtrA2 activation by the PDZ-binding hexapeptide (p6) exists at low temperatures only. This leads to a hypothesis that the structural changes induced by temperature and by peptides may be similar, as proposed previously by Martins et al. (2003).

## Electronic supplementary material

Below is the link to the electronic supplementary material.

Fig. S1 Changes of the emission maxima and bimolecular quenching constants of HtrA2 variants with temperature. **A** Emission maxima (*λ*_em_max) of HtrA2 variants with single-tryptophan substitutions. **B** Quenching of fluorescence of the tryptophan side chains of HtrA2 variants, presented as bimolecular quenching constants (*k*_*q*_). The data are as in Table S2 (*λ*_em_max) and Table 1 (*k*_*q*_). For clarity, only the values at temperatures 20 and 45 °C are shown (JPEG 90 kb)

High Resolution Image (TIFF 574 kb)

Table S1 Primers used to construct the *HtrA2* gene mutants. The exchanged nucleotides are marked in bold format (DOC 43 kb)

Table S2 Comparison of the fluorescence properties of the Trp residues in HtrA2 protein variants (DOC 106 kb)

Table S3 Thermal stability of the HtrA2 protein variants (DOC 34 kb)
