# Supplementary figures and images for: Temperature-induced changes of HtrA2(Omi) protease activity and structure
Source: Cell Stress Chaperones. 2012 Aug 1;18(1):35–51. doi: 10.1007/s12192-012-0355-1 (PMC3508124; doi:10.1007/s12192-012-0355-1)

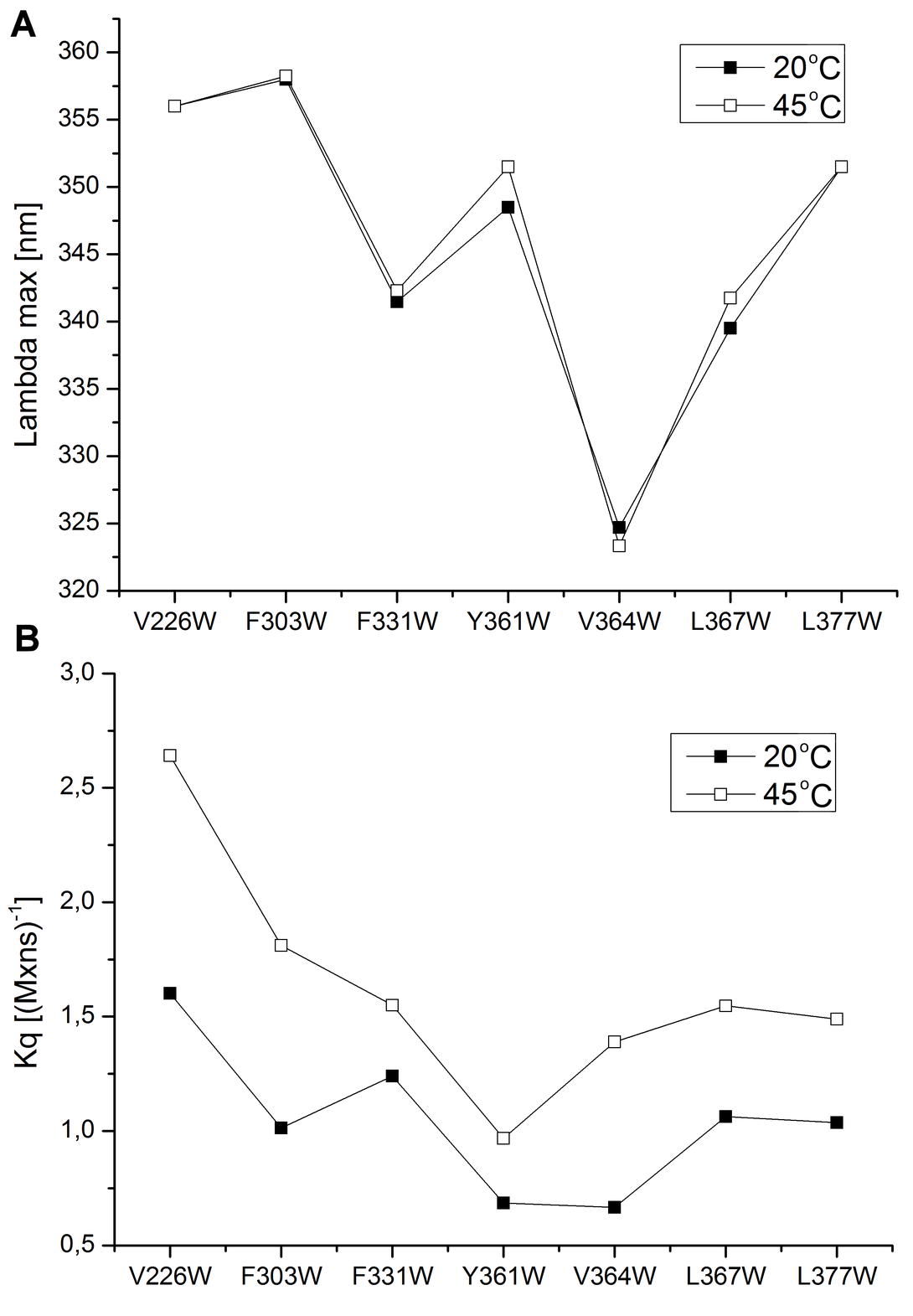

Supplement: Supplementary file 1 — Changes of the emission maxima and bimolecular quenching constants of HtrA2 variants with temperature. A Emission maxima (λ emmax) of HtrA2 variants with single-tryptophan substitutions. B Quenching of fluorescence of the tryptophan side chains of HtrA2 variants, presented as bimolecular quenching constants (k q). The data are as in Table S2 (λ emmax) and Table 1 (k q). For clarity, only the values at temperatures 20 and 45 °C are shown (JPEG 90 kb) [file 12192_2012_355_Fig7_ESM.jpg]

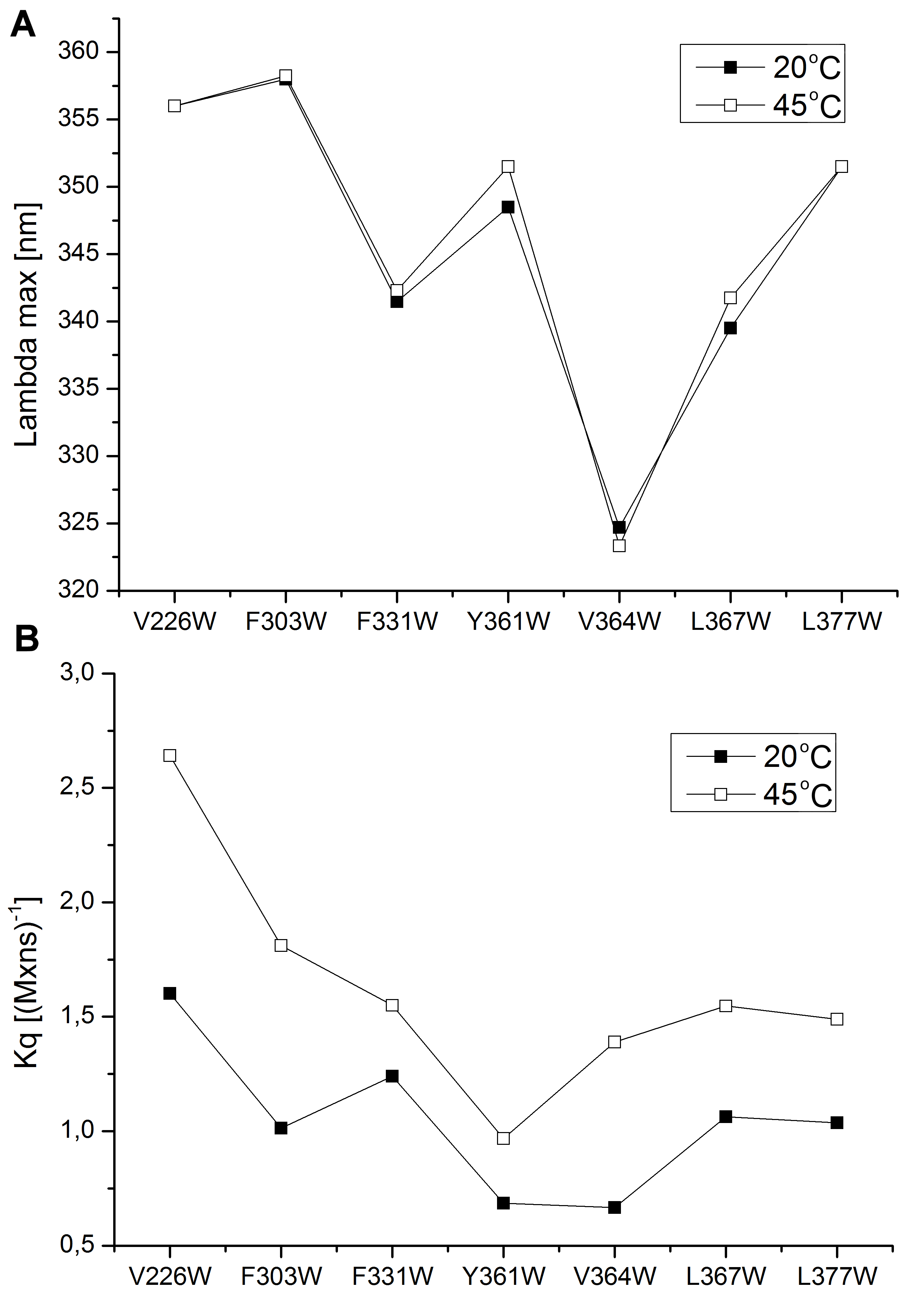

Supplement: Supplementary file 2 — High Resolution Image (TIFF 574 kb) [file 12192_2012_355_MOESM1_ESM.tif]
