# Supplementary material for: Temperature-induced changes of HtrA2(Omi) protease activity and structure
Source: Cell Stress Chaperones. 2012 Aug 1;18(1):35–51. doi: 10.1007/s12192-012-0355-1 (PMC3508124; doi:10.1007/s12192-012-0355-1)
Supplement: Supplementary file 3 — Primers used to construct the HtrA2 gene mutants. The exchanged nucleotides are marked in bold format (DOC 43 kb) [file 12192_2012_355_MOESM2_ESM.doc]

**Table S1.** Primers used to construct the *HtrA2* gene mutants. The exchanged nucleotides are marked in bold format

| Mutation | Oligonucleotide sequence (5’-3’) |
| --- | --- |
| V226K | CACAGCTGTGGATCCC**AA**GGCAGACATCGCAACG  CGTTGCGATGTCTGCC**TT**GGGATCCACAGCTGTG |
| V226W | CACAGCTGTGGATCCC**TG**GGCAGACATCGCAACG  CGTTGCGATGTCTGCC**CA**GGGATCCACAGCTGTG |
| F303W S306A | GCAGCTATTGATT**GG**GGAAAC**G**C**C**GGAGGTCC  GGACCTCC**G**G**C**GTTTCC**CC**AATCAATAGCTGC |
| S306A | GCTATTGATTTTGGAAAC**G**C**C**GGAGGTCCCCTGG  CCAGGGGACCTCC**G**G**C**GTTTCCAAAATCAATAGC |
| V325D | GTGAACACCATGAAGG**A**CACAGCTGGAATCTCC  GGAGATTCCAGCTGTG**T**CCTTCATGGTGTTCAC |
| I329N | CCATGAAGGTCACAGCTGGAA**A**CTCCTTTGCCATCC  GGATGGCAAAGGAG**T**TTCCAGCTGTGACCTTCATGG |
| F331Y | CAGCTGGAATCTCCT**A**TGCCATCCCTTCTGATC  GATCAGAAGGGATGGCA**T**AGGAGATTCCAGCTG |
| F331W | CAGCTGGAATCTCCT**GG**GCCATCCCTTCTGATC  GATCAGAAGGGATGGC**CC**AGGAGATTCCAGCTG |
| Y361W | TCCCAGCGGCGCT**GG**ATTGGGGTGATGATGC  GCATCATCACCCCAAT**CC**AGCGCCGCTGGGA |
| V364W | CGGCGCTACATTGGG**TG**GATGATGCTGACCCTG  CAGGGTCAGCATCATC**CA**CCCAATGTAGCGCCG |
| L367W | ATTGGGGTGATG**TG**GACCCTGAGTCCCAGC  GCTGGGACTCAGGGTC**CA**CATCATCACCCCAAT |
| L377W | CCAGCATCCTTGCTGAA**TGG**CAGCTTCGAGAACCAAGC  GCTTGGTTCTCGAAGCTG**CCA**TTCAGCAAGGATGCTGG |
| E429L | CTGAAGATGTTTAT**CT**AGCTGTTCGAACCCAATCCCAGTTGG  CCAACTGGGATTGGGTTCGAACAGCT**AG**ATAAACATCTTCAG |
| R432L | GCTGAAGATGTTTATGAAGCTGTT**TT**AACCCAATCCCAGTTGGC  GCCAACTGGGATTGGGTT**AA**AACAGCTTCATAAACATCTTCAGC |
