# Supplementary material for: Temperature-induced changes of HtrA2(Omi) protease activity and structure
Source: Cell Stress Chaperones. 2012 Aug 1;18(1):35–51. doi: 10.1007/s12192-012-0355-1 (PMC3508124; doi:10.1007/s12192-012-0355-1)
Supplement: Supplementary file 4 — Comparison of the fluorescence properties of the Trp residues in HtrA2 protein variants (DOC 106 kb) [file 12192_2012_355_MOESM3_ESM.doc]

**Table S2.** Comparison of the fluorescence properties of the Trp residues in HtrA2 protein variants

|  | τ_1_  [ns] | f_1_  [%] | τ_2_  [ns] | f_2_  [%] | τ_3_  [ns] | f_3_  [%] | τ_0_  [ns] | λ_em_max  [nm] |
| --- | --- | --- | --- | --- | --- | --- | --- | --- |
| W226 |  |  |  |  |  |  |  |  |
| 20 °C | 2.77±0.72 | 9.8 | 7.68±0.06 | 90.2 |  |  | 7.2±0.13 | 356±0.5 |
| 25 °C | 2.81±0.3 | 11.9 | 7.43±0.07 | 88.1 |  |  | 6.88±0.1 | 356.17±0.23 |
| 30 °C | 2.66±0.15 | 12.26 | 7.17±0.04 | 87.74 |  |  | 6.62±0.05 | 356.25±0.25 |
| 35 °C | 2.47±0.5 | 16.89 | 6.8±0.08 | 83.11 |  |  | 6.07±0.15 | 356.33±0.23 |
| 40 °C | 2.54±0.5 | 21.87 | 6.53±0.03 | 78.13 |  |  | 5.66±0.04 | 356.38±0.21 |
| 45 °C | 2.43±0.49 | 30.07 | 6.19±0.06 | 69.93 |  |  | 5.07±0.19 | 356±0.16 |
| W303 |  |  |  |  |  |  |  |  |
| 20 °C | 3.5±0.25 | 14.06 | 8.81±0.05 | 85.94 |  |  | 8.07±0.08 | 358±0.5 |
| 25 °C | 3.34±0.23 | 15.29 | 8.61±0.06 | 84.71 |  |  | 7.81±0.09 | 358±0.4 |
| 30 °C | 3.19±0.15 | 16 | 8.36±0.04 | 84 |  |  | 7.53±0.06 | 358±0 |
| 35 °C | 2.87±0.64 | 17.48 | 8.09±0.05 | 82.52 |  |  | 7.18±0.15 | 358±0 |
| 40 °C | 2.87±0.1 | 18.96 | 7.82±0.04 | 81.04 |  |  | 6.88±0.06 | 358.13±0.21 |
| 45 °C | 2.75±0.32 | 20.84 | 7.49±0.05 | 79.16 |  |  | 6.5±0.1 | 358.25±0.21 |
| W331 |  |  |  |  |  |  |  |  |
| 20 °C | 0.56±0.08 | 5.75 | 3.44±0.15 | 50.5 | 7.02±0.14 | 43.75 | 4.84±0.14 | 341.48±0.2 |
| 25 °C | 0.67±0.09 | 7.77 | 3.69±0.18 | 61.72 | 7.73±0.42 | 30.51 | 4.69±0.25 | 341.64±0.25 |
| 30 °C | 0.76±0.07 | 9.97 | 3.77±0.13 | 67.57 | 8.44±0.46 | 22.46 | 4.52±0.2 | 341.56±0.45 |
| 35 °C | 0.92±0.05 | 13.2 | 3.85±0.1 | 70.81 | 9.46±0.5 | 16 | 4.36±0.16 | 341.77±1.05 |
| 40 °C | 1.00±0.07 | 16.02 | 3.76±0.16 | 69.38 | 9.82±1.57 | 14.6 | 4.2±0.35 | 341.74±0.2 |
| 45 °C | 0.87±0.07 | 15.59 | 3.48±0.11 | 68.89 | 9±0.47 | 15.53 | 3.93±0.16 | 342.29±1 |
| W361 |  |  |  |  |  |  |  |  |
| 20 °C | 0.81±0.26 | 9.34 | 3.73±0.24 | 61.39 | 8.99±0.68 | 30.3 | 5.07±0.38 | 348.5±0.4 |
| 25 °C | 0.91±0.1 | 11.74 | 3.74±0.08 | 63.64 | 9.67±0.13 | 26.16 | 5.02±0.1 | 350.5±0.5 |
| 30 °C | 0.86±0.1 | 11.11 | 3.49±0.16 | 61.8 | 9.16±0.64 | 27.94 | 4.8±0.29 | 350.5±0.5 |
| 35 °C | 0.82±0.09 | 12.46 | 3.43±0.12 | 61.73 | 9.76±0.25 | 26.77 | 4.83±0.15 | 351±0.35 |
| 40 °C | 0.77±0.21 | 12.74 | 3.27±0.2 | 59.8 | 9.26±0.88 | 28.11 | 4.64±0.4 | 351.5±0.25 |
| 45 °C | 0.75±0.19 | 13.6 | 3.17±0.18 | 58.61 | 9.21±0.1 | 28.45 | 4.56±0.41 | 351.5±0 |
| W364 |  |  |  |  |  |  |  |  |
| 20 °C | 0.74±0.2 | 14.97 | 2.94±0.12 | 72.88 | 7.58±0.55 | 12.37 | 3.18±0.18 | 324.7±0.6 |
| 25 °C | 0.8±0.22 | 16.51 | 2.93±0.13 | 73.36 | 7.96±0.82 | 9.99 | 3.06±0.21 | 324.5±0.35 |
| 30 °C | 0.73±0.06 | 14.7 | 2.74±0.05 | 74.9 | 7.57±0.26 | 9.96 | 2.91±0.07 | 326.25±0.35 |
| 35 °C | 0.72±0.2 | 17.06 | 2.67±0.1 | 74.13 | 7.96±0.59 | 8.59 | 2.78±0.16 | 325.75±0.5 |
| 40 °C | 0.71±0.05 | 16.75 | 2.5±0.06 | 74.28 | 7.79±0.36 | 8.51 | 2.64±0.08 | 324±1.17 |
| 45 °C | 0.78±0.2 | 22.37 | 2.51±0.08 | 70.29 | 8.55±0.41 | 6.59 | 2.5±0.13 | 323.33±0.35 |
| W367 |  |  |  |  |  |  |  |  |
| 20 °C | 0.63±0.07 | 11.88 | 3.04±0.11 | 62.51 | 7.48±0.2 | 26.45 | 3.95±0.13 | 339.5±0.5 |
| 25 °C | 0.66±0.11 | 13.3 | 2.93±0.12 | 62.05 | 7.42±0.22 | 25.33 | 3.78±0.15 | 339.2±1.02 |
| 30 °C | 0.59±0.07 | 13.95 | 2.79±0.09 | 61.35 | 7.17±0.18 | 25.83 | 3.64±0.11 | 340.17±0.23 |
| 35 °C | 0.67±0.08 | 15.55 | 2.79±0.11 | 60.89 | 7.27±0.22 | 23.77 | 3.53±0.11 | 341±0.5 |
| 40 °C | 0.61±0.07 | 15.93 | 2.68±0.1 | 60.79 | 7.1±0.2 | 23.97 | 3.43±0.13 | 340±0.5 |
| 45 °C | 0.62±0.05 | 17.03 | 2.62±0.06 | 59.55 | 6.92±0.14 | 23.88 | 3.32±0.08 | 341.75±0.84 |
| W377 |  |  |  |  |  |  |  |  |
| 20 °C | 2.52±0.03 | 20.6 | 7.76±0.04 | 79.4 |  |  | 6.68±0.1 | 351.5±0.48 |
| 25 °C | 2.42±0.03 | 21.47 | 7.53±0.04 | 78.53 |  |  | 6.43±0.1 | 351.5±0.7 |
| 30 °C | 2.26±0.06 | 21.86 | 7.31±0.04 | 78.14 |  |  | 6.21±0.05 | 351.5±0.5 |
| 35 °C | 2.24±0.04 | 22.19 | 7.11±0.04 | 77.81 |  |  | 6.03±0.11 | 351.5±0.5 |
| 40 °C | 2.08±0.06 | 22.64 | 6.89±0.03 | 77.36 |  |  | 5.8±0.04 | 352.5±1.1 |
| 45 °C | 2.1±0.03 | 23.7 | 6.66±0.04 | 76.3 |  |  | 5.58±0.09 | 351.5±0.25 |

The fluorescence maxima (λ_em_max) and fluorescence decay of the HtrA2 single-Trp variants were measured at different temperatures, without quencher. The lifetimes (τ_1_ , τ_2_ and τ_3_) of the excited Trp residues were calculated as described in the Materials and Methods section. The τ_o_  values are the mean fluorescence lifetimes.
