# Supplementary material for: Temperature-induced changes of HtrA2(Omi) protease activity and structure
Source: Cell Stress Chaperones. 2012 Aug 1;18(1):35–51. doi: 10.1007/s12192-012-0355-1 (PMC3508124; doi:10.1007/s12192-012-0355-1)
Supplement: Supplementary file 5 — Thermal stability of the HtrA2 protein variants (DOC 34 kb) [file 12192_2012_355_MOESM4_ESM.doc]

**Table S3. Thermal stability of the HtrA2 protein variants**

| HtrA2 protein | T_m_ [^o^C] | Difference between  HtrA2 variant  and HtrA2S306A [^o^C] |
| --- | --- | --- |
| S306A | 70,71 | 0 |
| S306A V226K | 68,67 | -2,04 |
| S306A Y361W | 69,88 | -0,83 |
| S306A I329N | 70,75 | +0,04 |
| S306A F303W | 72,19 | +1,48 |
| S306A V325D | 67,39 | -3,32 |
| S306A L367W | 69,84 | -0,87 |
| S306A F331Y | 72,47 | +1,78 |
| S306A F331W | 70,52 | -0,19 |
| S306A L377W | 70,22 | -0,49 |
| S306A V364W | 71,31 | +0,60 |
| S306A V226W | 68,63 | -2,08 |
| S306A R432L | 70,44 | -0.27 |

The far-UV circular dichroism (CD) spectra of the HtrA2 variants were recorded at temperatures 20-85 °C, in the range of 200-260 nm. The melting point temperatures (T_m_) were calculated from CD signals recorded at 207 nm, as described in the Materials and Methods section.
